# Neural Field Theory of Evoked Response Sequences and Mismatch Negativity With Adaptation

**DOI:** 10.3389/fnhum.2021.655505

**Published:** 2021-08-16

**Authors:** Peter A. Robinson, Natasha C. Gabay, Tara Babaie-Janvier

**Affiliations:** ^1^School of Physics, University of Sydney, Sydney, NSW, Australia; ^2^Center of Excellence for Integrative Brain Function, University of Sydney, Sydney, NSW, Australia

**Keywords:** evoked response, mismatch negativity, neural field theory, adaptation, modeling

## Abstract

Physiologically based neural field theory of the corticothalamic system is used to calculate the responses evoked by trains of auditory stimuli that correspond to different cortical locations via the tonotopic map. The results are shown to account for standard and deviant evoked responses to frequent and rare stimuli, respectively, in the auditory oddball paradigms widely used in human cognitive studies, and the so-called mismatch negativity between them. It also reproduces a wide range of other effects and variants, including the mechanism by which a change in standard responses relative to deviants can develop through adaptation, different responses when two deviants are presented in a row or a standard is presented after two deviants, relaxation of standard responses back to deviant form after a stimulus-free period, and more complex sequences. Some cases are identified in which adaptation does not account for the whole difference between standard and deviant responses. The results thus provide a systematic means to determine how much of the response is due to adaptation in the system comprising the primary auditory cortex and medial geniculate nucleus, and how much requires involvement of higher-level processing.

## 1. Introduction

**Figure 1:**
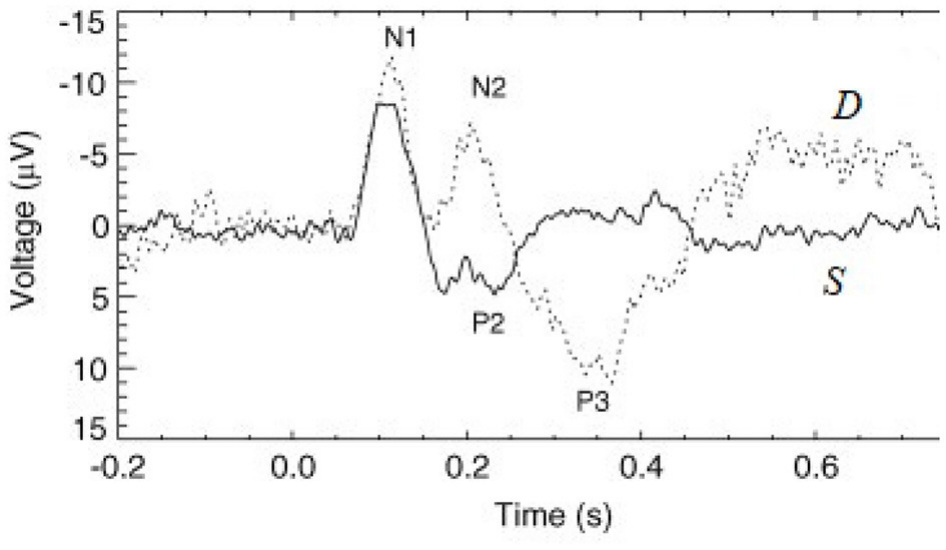
Examples of typical standard (*S*, solid line) and deviant (*D*, dashed line) evoked responses to auditory stimuli in an auditory oddball experiment (Kerr et al., 2009). The traditional phenomenological “components” (peaks and troughs) are labeled, where N1 and P2 indicate a negative deflection around 100 ms and a positive one around 200 ms, respectively, for example.

Evoked responses (ERs) to short impulse-like stimuli are commonly used to probe human cognitive processes. These responses are usually measured using electroencephalography (EEG) or magnetoencephalography (MEG), most often in auditory experiments, although other sensory modalities are also used (Luck and Kappenman, 2011; Niedermeyer and Lopes da Silva, 2011; Luck, 2014). Trains of ERs show a rich repertoire of effects in response to any violation of regularity—changes in frequency, location, duration, and intensity (Näätänen, 2003; Luck and Kappenman, 2011; Luck, 2014). Such effects are most often elicited in so-called oddball paradigms in which frequent standard (S) stimuli are interspersed with rarer deviants (D), which elicit very different ERs in general, so long as they are discriminable (Sams et al., 1985; Näätänen, 2003; Garrido et al., 2013), as seen in Figure 1. Deviants that are only marginally discriminable give an intermediate response (Sams et al., 1985; Garrido et al., 2009a). Here and throughout this paper we denote stimuli with calligraphic font to distinguish them from responses, which are written in italic font.

Deviant responses *D* are believed to reflect a response to novelty and, indeed, at the beginning of a stimulus train, all stimuli evoke *D* responses, but standard (*S*) responses evolve over a few presentations to their limiting form *S*_∞_ as their preponderance becomes established (Näätänen, 2003; Garrido et al., 2009a, 2013; Luck and Kappenman, 2011; Luck, 2014); since a typical interstimulus interval is ~1 s, this sets an adaptation timescale of several seconds. Similarly, after a pause in stimulation, *S* responses return to the *D* form over a few seconds, again indicating that the effects responsible for the difference between the two have a lifetime of seconds (Cowan, 1984; Winkler et al., 1993; Loveless et al., 1996; Näätänen, 2003). Likewise, both *D* and *S* responses differ from their usual forms if two D stimuli occur consecutively or an S follows two Ds (Sams et al., 1984). This implies that the different responses at least partly reflect recent stimuli rather than their long-term average probabilities.

At a more complex level, a *D* response is seen to a repeated tone in a descending sequence of tones, where there is no repeated S stimulus (Näätänen et al., 1989a; Tervaniemi et al., 1994; Näätänen, 2003; Garrido et al., 2009b, 2013); likewise, a *D* response occurs after a stimulus that is omitted or changed in duration or intensity (Näätänen et al., 1989b, 2007; Yabe et al., 1997; Näätänen, 2003; Salisbury, 2012); and, more abstract, high-level, irregularities such as violations of grammatical categories or phonemic regularities in a sequence can elicit *D* responses (May et al., 1999; Nelken, 2004; Garrido et al., 2013). Frequency-deviant and random-frequency stimuli elicit an increasing proportion of *D* responses as the overall frequency range of the ensemble of stimuli increases well beyond the discriminability threshold (Sams et al., 1985; Garrido et al., 2013) and it has been argued that the brain can thus encode information about the statistical distribution of stimuli (Garrido et al., 2013).

The difference between *S* and *D* responses is often quantified via the mismatch negativity (MMN), which is mathematically constructed by subtracting the *S* response from the *D* one (Näätänen, 2003; Luck and Kappenman, 2011; Luck, 2014). A longstanding debate is whether the MMN (i) reflects differences in responses due to adaptation of the primary sensory system (relevant thalamic relay nuclei and primary sensory cortex, although few references discuss thalamic participation) to repeated stimuli, with the part of the system that processes S stimuli being driven further from its starting parameters than the part that processes D stimuli (Jääskeläinen et al., 2004; Näätänen et al., 2005; Garrido et al., 2009b; May et al., 2015); (ii) a reflection of separate, possibly memory-related or internal-model dependent, stimulus-comparison processes in primary cortex or higher-order cortical areas (Atienza et al., 2001; Näätänen, 2003; Jääskeläinen et al., 2004; Garrido et al., 2009b); or (iii) a combination of both adaptation and stimulus-comparison. The more abstract cases of *D* responses appear to point to the latter interpretation (Näätänen, 2003; Näätänen et al., 2005; Garrido et al., 2009b), but basic biophysics, the evolution of *S* and *D* responses during long trains, the decay of their distinction during a few-second stimulation pause, and the existence of MMN in coma imply a role for the former explanation (Schröger, 1998; Näätänen, 2003; Jääskeläinen et al., 2004; Sussman et al., 2014; May et al., 2015). In this work we take the viewpoint that both types of mechanisms are likely to be simultaneously in play, so the issue we address is which cases can be accounted for by adaptation—potentially the other, more abstract, cases then involve higher cortical areas in further processing and top-down feedback. We note that adaptation is likely to be involved in responses of nonhuman animals without involvement of language processing, as exemplified by stimulus specific adaptation studied in rats Malmierca et al. (2009), Szymanski et al. (2009), Pérez-González and Malmierca (2014); however, we restrict attention to parameters appropriate to humans in the present work.

A weakness of traditional phenomenological analyses of ER time series is that they are usually recorded at hundreds of samples per second, but quantified by noting only the amplitudes and timings of a few peaks and troughs in the waveform, or of underlying “components” that sum to produce them (Luck and Kappenman, 2011; Luck, 2014); each component is asserted to be produced by a particular “generator” with a given location and sign, normally assumed to correspond to a group of excitatory or inhibitory neurons that respond with a fixed post-stimulus delay and temporal profile (Luck and Kappenman, 2011; Luck, 2014). Hence, the MMN is often assumed to correspond to a separate component and corresponding underlying set of MMN neurons. Whilst components have characteristic timings and associated spatial structures (Luck, 2014), this procedure commonly commences analysis by omitting almost all the data points that have been recorded, which is a questionable procedure to include as a key step in any data-analysis pipeline, especially as it makes component timings very sensitive to noise near extremums. Moreover, it has been shown that these timings evolve with age, and the extremums even invert polarity during development (Kerr et al., 2010), so the very use of timings and polarities to designate features is problematic in itself. For example, use of traditional components has tended to limit adaptation theories to qualitative conclusions that particular components are attenuated by adaptation without changing their timing or polarity (Näätänen et al., 2007; Garrido et al., 2009b).

Once it is recognized that the brain is a physical system, whose dynamics generate EEG and MEG signals, including ERs, new analysis and modeling avenues are opened and it is quickly revealed that components are not fundamental building blocks of the dynamics (Freeman, 1975; Rennie et al., 2002; Kerr et al., 2008, 2011); rather they reflect damped physical oscillations of brain activity in natural modes (Demiralp et al., 1998; Rennie et al., 2002; van Albada et al., 2010; Başar, 2012; Mukta et al., 2019; Babaie-Janvier and Robinson, 2020). In particular, it has long been noted that these signals depend on the average responses of large numbers of neurons that are detected by a given electrode or coil (Nunez, 1995; Nunez and Srinivasan, 2006; Niedermeyer and Lopes da Silva, 2011).

Neural field theory (NFT) has been developed by many authors to predict mean neural activity at scales of tenths of a millimeter and above by starting from physiological and anatomical parameters (Beurle, 1956; Wilson and Cowan, 1972, 1973; Nunez, 1974, 1995; Freeman, 1975; Lopes da Silva et al., 1976; Amari, 1977; Wright and Liley, 1994; Jirsa and Haken, 1996; Steyn-Ross et al., 1999; Robinson et al., 2002, 2004; Deco et al., 2008; Bressloff, 2012; Coombes et al., 2014; Sanz-Leon et al., 2018). In normal regimes of moderate activity, measurable signals have been shown to be approximately linearly related to perturbations of underlying neural activity from its overall mean (Nunez, 1995; Robinson et al., 1997; Deco et al., 2008). In particular, ERs reflect the activity produced by near-impulsive stimuli and the conditions of the brain that generate them can be inferred by fitting model predictions to data (Rennie et al., 2002; Kerr et al., 2011). Most significantly, the strengths of connections, or gains, between excitatory and inhibitory populations in the cortex and thalamus prove to be primary determinants of the forms of ERs and other activity phenomena (Rennie et al., 2002; Kerr et al., 2008, 2011; van Albada et al., 2010; Babaie-Janvier and Robinson, 2020). For reviews of NFT and its use in a wide range of contexts see Deco et al. (2008), Coombes et al. (2014), and Sanz-Leon et al. (2018) for example.

NFT impulse-response models of *S* and *D* ERs have been successfully fitted to data from cohorts of up to 1,500 subjects (Kerr et al., 2011). Notably, the inferred prestimulus parameters for *S* and *D* responses prove to be quite different from one another, and from those of background EEG (van Albada et al., 2010; Kerr et al., 2011). NFT has since been used to analyze the dynamics of stimulus prediction and automatic attention in the corticothalamic system (Babaie-Janvier and Robinson, 2018, 2019, 2020). This work showed that stimulus-driven gain changes due to a variety of processes such as adaptation and facilitation can improve prediction by increasing the gains that relate to salient stimuli, thereby implementing a form of attention and providing a basis to progress to higher order cognitive processes such as top-down feedback within the cortex (Gazzaley et al., 2005; Friston, 2010, 2011; Babaie-Janvier and Robinson, 2020). Moreover, it has been shown that *S* and *D* responses can be separately reproduced as impulse responses from the background EEG state when attentional gain dynamics is taken into account (Babaie-Janvier and Robinson, 2019, 2020). These results have also been interpreted in terms of engineering control theory (Ogata and Yang, 1970; Freeman, 1975; Babaie-Janvier and Robinson, 2020). In these physically based approaches the building blocks of responses are the same damped corticothalamic oscillations that account for ongoing EEG characteristics and other phenomena.

In the present work we develop a unified NFT theory, including adaptation, that can account for S and D responses to sequences of simple stimuli, including development of distinct response characteristics. This both simplifies and reduces the number of parameters required and enables a wide range of experimental conditions to be reproduced from a single model. Moreover, it predicts the entire waveform, not just peaks and troughs, and incorporates changes in amplitudes and timings of oscillations due to changes in corticothalamic parameters. It can thus potentially be fitted to experiment to determine brain-state parameters, as has been done with prior variants (Kerr et al., 2011; Babaie-Janvier and Robinson, 2020).

The structure of this paper is as follows: In section 2, we generalize our prior NFT model to calculate corticothalamic transfer functions and resulting ERs to arbitrary stimuli in the absence of higher-order cognitive processes, but incorporating adaptation effects and stimulus feature dependence. In section 3, we calibrate the model parameters by requiring that it reproduce *D* responses at the background EEG baseline state, and *S* responses when repetitively driven by impulsive stimuli that move the system away from the background state via adaptation. This provides the basis to analyze responses to arbitrary stimulus sequences. In the remainder of section 3, we test the theory's predictions for a range of experimental sequences to begin to explore which phenomena can be explained by adaptation and which likely require higher-order processing, but we stress that the literature is too vast to address all possibilities in the present work. Section 4 summarizes the main findings and outlines directions for future work.

## 2. Materials and Methods

In this section we first review how ERs represent impulse responses of a linear approximation to brain dynamics and that these are directly related to system transfer functions (Freeman, 1975; Rennie et al., 2002; Kerr et al., 2008, 2011; Babaie-Janvier and Robinson, 2020). This approach has proved to be successful in the past, and has been extensively tested against experimental results (Kerr et al., 2011). We then briefly review the existing NFT corticothalamic model that is used in the analysis and generalize its dynamics to include slow adaptation processes that reflect a “memory trace.” A feature map such as the tonotopic map in auditory cortex is then incorporated.

### 2.1. ERs as Transfer Functions

Cortical evoked responses (ERs) and magnetoencephalographic (MEG) analogs are generated primarily by perturbations in the activity ϕ_*e*_ of pyramidal excitatory cells due to dynamics in the corticothalamic system (Nunez, 1995). In the simplest approximation, we can write

(1) $$\begin{array}{l} \phi_{e}^{\left(1\right)}\left(t\right)=\int_{-\infty }^{t}T\left(t-t^{\prime }\right)\phi_{n}^{\left(1\right)}\left(t^{\prime }\right)dt^{\prime }, \end{array}$$

for a purely temporal response, where *T* is the system linear response function, which is zero for *t* < *t*′ to preserve causality, and $\phi_{n}^{\left(1\right)}$ is the incoming non-corticothalamic stimulus to the corticothalamic system. The form in Equation (1) can be generalized to include spatial aspects but here we focus on the temporal domain in order to bring out the main aspects without undue complexity; generalization to include multiple spatial eigenmodes can be carried out in a similar way (Kerr et al., 2008; Mukta et al., 2019). Equation (1) can be Fourier transformed to yield

(2) $$\begin{array}{l} \phi_{e}^{\left(1\right)}\left(\omega \right)=T\left(\omega \right)\phi_{n}^{\left(1\right)}\left(\omega \right), \end{array}$$

If the input in (1) is a delta function $\phi_{n}\left(t^{\prime }\right)=\delta \left(t^{\prime }-t_{0}\right)$, one finds

(3) $$\begin{array}{l} \phi_{e}^{\left(1\right)}\left(t\right)=T\left(t-t_{0}\right), \end{array}$$

whence we see that the ER to a delta input and the transfer function are one and the same. More generally, subsequent physical phenomena such as volume conduction, measurement effects, and post-processing also need to be taken into account in the overall transfer function from stimulus to measurement, but we omit discussion of these issues for simplicity because they do not strongly affect the time course of ERs, which is our main focus here.

In general, the transfer function itself can be changed by the stimulus, owing to a variety of fast and slow dynamical effects (Koch, 1999; Rennie et al., 1999, 2000, 2002; Robinson and Roy, 2015; Babaie-Janvier and Robinson, 2019), but to treat such effects, we must first introduce neural field theory and a model of the corticothalamic system.

### 2.2. Neural Field Theory of the Corticothalamic System

The baseline model that we generalize in the present work has been developed and successfully applied over many years, as mentioned in section 1. The specific formulation used here is the one from Babaie-Janvier and Robinson (2019, 2020), which we outline and generalize. Note that some of the descriptions of model elements in sections 2.2 and 2.3 are identical to those in these prior works to avoid introducing errors and ambiguities by changing the wording simply for the sake of change.

**Figure 2:**
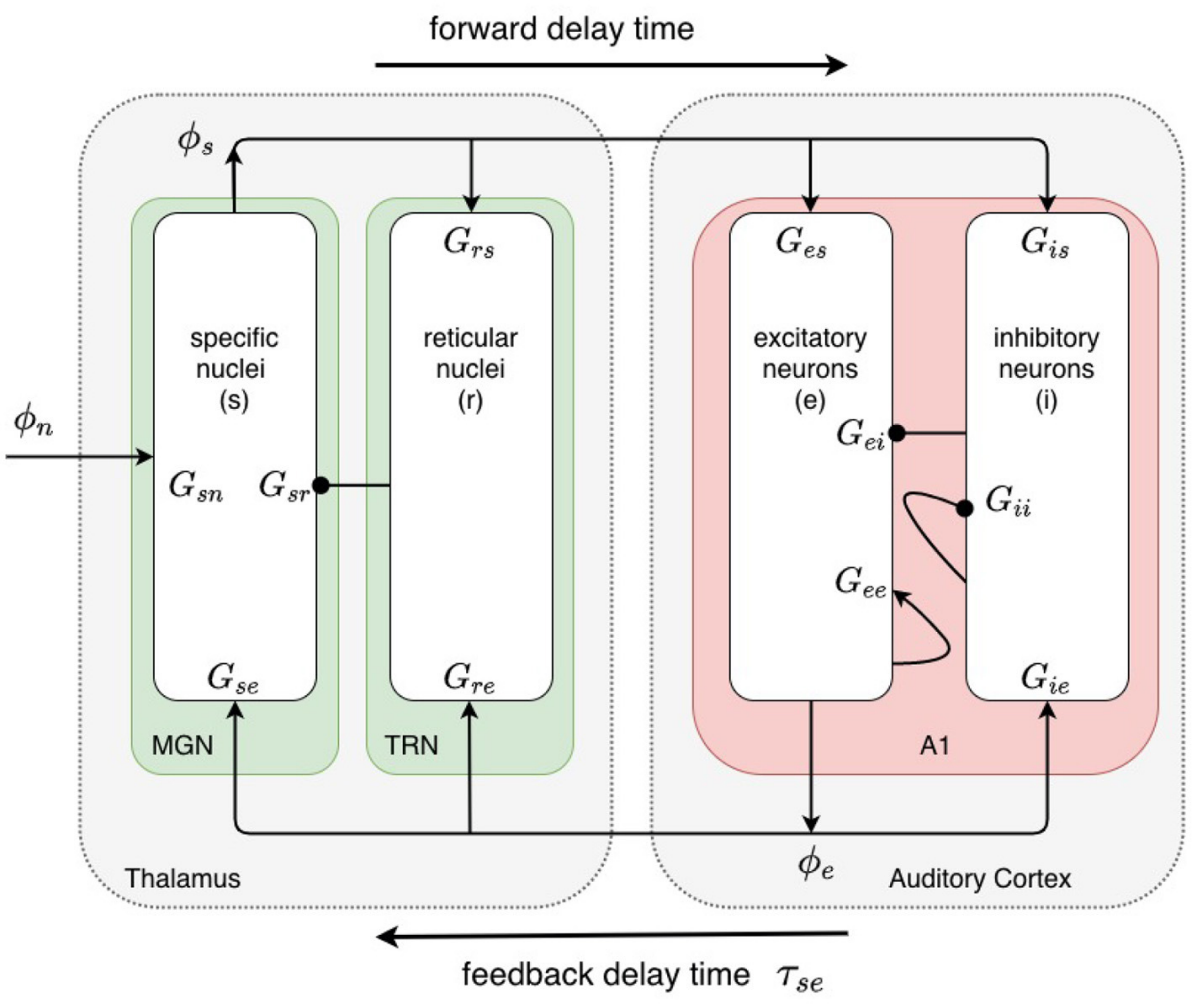
Physiologically based corticothalamic neural field model. Arrowheads represent excitatory effects while circles depict inhibitory ones. The populations are cortical excitatory (*e*) and inhibitory (*i*) neurons, the thalamic reticular nucleus (TRN, *r*), thalamic relay neurons in the medial geniculate nucleus (MGN, *s*) that project to the primary auditory cortex (A1), and non-corticothalamic neurons responsible for external inputs (*n*).

The baseline model, shown in Figure 2, incorporates the cortex and thalamus and their connectivities; each includes distinct populations of neurons: cortical excitatory pyramidal (*e*) and short-range mostly inhibitory (*i*) neurons, the thalamic reticular nucleus (TRN) (*r*), thalamic relay neurons (*s*), and non-corticothalamic neurons that provide external inputs (*n*). In this study, the relevant relay nucleus is the medial geniculate nucleus, whose projections are to primary auditory cortex. Excitatory projections to the TRN exist from thalamocortical feedforward axons and corticothalamic feedback axons, and there are inhibitory projections from the TRN onto thalamic relay neurons.

The state of each neural population *a*, is represented by the local mean cell-body potential *V*_*a*_(**r**, *t*) relative to resting, the mean firing rate *Q*_*a*_(**r**, *t*), and the outgoing axonal pulse rate field ϕ_*a*_(**r**, *t*). NFT averages over spatial scales below a few tenths of a millimeter to obtain equations for evolution of these dynamical variables (Wilson and Cowan, 1973; Freeman, 1975; Deco et al., 2008).

The mean firing rate *Q*_*a*_ has a sigmoidal response to increasing *V*_*a*_, which can be approximated as (Wilson and Cowan, 1973; Freeman, 1975; Deco et al., 2008)

(4) $$\begin{array}{l} Q_{a}\left(\text{r},t\right)=S\left[V_{a}\left(\text{r},t\right)\right]=\frac{Q_{\text{max}}}{1+\exp\left\{-\left[V_{a}\left(\text{r},t\right)-\theta \right]/\sigma^{\prime }\right\}}, \end{array}$$

where θ is the mean neural firing threshold and $\sigma^{\prime }\pi /\sqrt{3}$ is the standard deviation of the difference between the steady state soma voltage of individual neurons and their thresholds.

The potential *V*_*a*_(**r**, *t*) results from all afferent neural synaptic receptors of types *b* and is given by

(5) $$\begin{array}{l} {\hat{D}}_{\alpha }\left(t\right)V_{a}\left(\text{r},t\right)=\underset{b}{\sum }N_{ab}s_{ab}\left(\text{r},t\right)\phi_{b}\left(\text{r},t-\tau_{ab}\right), \end{array}$$

(6) $$\begin{array}{l} {\hat{D}}_{\alpha }\left(t\right)=\frac{1}{\alpha \beta }\frac{d^{2}}{dt^{2}}+\left(\frac{1}{\alpha }+\frac{1}{\beta }\right)\frac{d}{dt}+1, \end{array}$$

where the differential operator ${\hat{D}}_{a}$ governs the temporal response of *V*_*ab*_ to afferent pulse rate fields ϕ_*b*_, encapsulating the rates β and α of the rise and fall, respectively, of the response at the cell body, which are assumed equal for all *ab* here; *N*_*ab*_ is the mean number of synapses on neurons *a* from neurons of type *b*; *s*_*ab*_ is the mean time-integrated strength of soma response per incoming spike; and ϕ_*b*_(**r**, *t* − τ_*ab*_) is the mean spike arrival rate from neurons *b*, delayed by τ_*ab*_ due to discrete anatomical separations between different populations. The overall connection strength to neural population *a* from *b* is

(7) $$\begin{array}{l} \nu_{ab}\left(\text{r},t\right)=N_{ab}s_{ab}\left(\text{r},t\right). \end{array}$$

Outgoing neural pulses within each population are averaged over short scales to form a field ϕ_*a*_(**r**, *t*) whose source is *Q*_*a*_(**r**, *t*). This field propagates at the characteristic axonal velocity *v*_*a*_ and approximately obeys the damped wave equation (Jirsa and Haken, 1996; Robinson et al., 1997),

(8) $$\begin{array}{l} {\hat{D}}_{a}\left(\text{r},t\right)\phi_{a}\left(\text{r},t\right)=Q_{a}\left(\text{r},t\right), \end{array}$$

(9) $$\begin{array}{l} {\hat{D}}_{a}\left(\text{r},t\right)=\frac{1}{\gamma_{a}^{2}}\frac{\partial^{2}}{\partial t^{2}}+\frac{2}{\gamma_{a}}\frac{\partial }{\partial t}+1-r_{a}^{2}\nabla^{2}, \end{array}$$

where the damping rate γ_*a*_ satisfies γ_*a*_ = *v*_*a*_/*r*_*a*_, where *r*_*a*_ is the characteristic range of axons *a*. In the corticothalamic system, only axons of excitatory cortical pyramidal neurons are long enough to cause significant propagation effects in Equation (9). In the other populations, we assume the axonal lengths are near zero (i.e., *r*_*a*_ ≈ 0) so $D_{a}\approx 1$ which results in ϕ_*a*_(**r**, *t*) = *Q*_*a*_(**r**, *t*) for these populations.

We set ν_*ie*_ = ν_*ee*_, ν_*ii*_ = ν_*ei*_, and ν_*is*_ = ν_*es*_ because the number of cortical synapses is very nearly proportional to the numbers of source and target neurons (Wright and Liley, 1996; Braitenberg and Schüz, 1998), assuming that synaptic types are determined by the source neurons. Forward time delays are τ_*es*_ = τ_*is*_ ≈ 20 ms for thalamocortical signals and feedback delays are τ_*se*_ = τ_*re*_ ≈ 60 ms for corticothalamic signals, while the other τ_*ab*_ are zero; time delays in the long-range excitatory axons in the cortex are included via Equation (9).

Table 1 lists nominal values of model parameters (Robinson et al., 2004) for resting EEG. These values were estimated for normal adults and they have been extensively used in the comparison with experiments, as mentioned in section 1.

**Table 1:** Estimated brain parameters for normal adult humans in the alert, eyes-open state.

**Quantity**	**Description**	**Resting EEG (P)**	**ER**	**Resting EEG**	**Unit**
*Q*_max_	Max firing rate	250	250	250	s^−1^
θ	Firing threshold	15	15	15	mV
σ′	Threshold spread	3.3	3.3	3.3	mV
γ_*e*_	Cortical damping rate	116	200	116	s^−1^
α	Inverse decay time	80	45	80	s^−1^
β	Inverse rise time	320	180	320	s^−1^
τ_*es*_	Forward delay time	20	32	20	ms
τ_*se*_	Feedback delay time	60	32	60	ms
$\phi_{e}^{\left(0\right)}$	Firing rate *e* neurons	16	16	16	s^−1^
$\phi_{s}^{\left(0\right)}$	Firing rate *s* neurons	16	16	16	s^−1^
$\phi_{r}^{\left(0\right)}$	Firing rate *r* neurons	16	16	16	s^−1^
$\phi_{n}^{\left(0\right)}$	Firing rate *n* neurons	16	16	16	s^−1^
ρ_*e*_	For *e* neurons	4,200	4,200	4,200	V^−1^ s^−1^
ρ_*s*_	For *s* neurons	4,200	4,200	4,200	V^−1^ s^−1^
ρ_*r*_	For *r* neurons	6,300	6,300	6,300	V^−1^ s^−1^
$G_{ee}^{\left(0\right)}$	Gain to *e* from *e*	5.9	3.1	6.8	−
$G_{se}^{\left(0\right)}$	Gain to *s* from *e*	2.5	1.18	2.5	−
$G_{ii}^{\left(0\right)}$	Gain to *i* from *i*	−8.1	−10.8	−8.1	−
$G_{sr}^{\left(0\right)}$	Gain to *s* from *r*	−1.9	−2.8	−1.9	−
$G_{es}^{\left(0\right)}$	Gain to *e* from *s*	1.7	0.74	1.7	−
$G_{sn}^{\left(0\right)}$	Gain to *s* from *n*	0.8	0.8	0.8	−
$G_{ie}^{\left(0\right)}$	Gain to *i* from *e*	5.9	3.1	6.8	−
$G_{re}^{\left(0\right)}$	Gain to *r* from *e*	1.3	3.4	1.0	−
$G_{ei}^{\left(0\right)}$	Gain to *e* from *i*	−8.1	−10.8	-8.1	−
$G_{rs}^{\left(0\right)}$	Gain to *r* from *s*	0.19	0.28	0.19	−
$G_{is}^{\left(0\right)}$	Gain to *i* from *s*	1.7	0.74	1.7	−

*The first two columns give the symbol and description of each quantity. The third column shows resting-EEG values corresponding to the P state; these are also used as the initial values for ERs in which the gains adjust dynamically as part of the response. The fourth column lists static-gain ER values adapted from Table 1 of Kerr et al. (2008), previously used to match standard ERs using static gains. The fifth columns lists resting-EEG values used in previous work (Babaie-Janvier and Robinson, 2020). The final column gives units*.

### 2.3. Corticothalamic Transfer Functions

The above NFT equations are nonlinear in general. By setting all spatial and temporal derivatives in these equations to zero, we find spatially uniform steady-states of the system, which are interpreted as characterizing the baseline of normal activity, with firing rates that are in accord with experiments (Robinson et al., 2002, 2004). Linear perturbations from these steady states represent time dependent brain activity by which numerous experimental phenomena have been reproduced, including evoked responses (Robinson et al., 1997, 2002, 2004, 2005; Rennie et al., 2002; O'Connor and Robinson, 2004; Kerr et al., 2008, 2011; van Albada et al., 2010; Roberts and Robinson, 2012; Abeysuriya et al., 2015).

#### 2.3.1. Perturbation Expansion

We expand the equations in section 2.2 to first order in perturbations relative to the steady state, denoting steady-state and perturbed quantities by the superscripts 0 and 1, respectively, and neglecting second- and higher-order terms. We also omit the **r** dependence from this point on, although its retention up to the present point was necessary to correctly account for the parameters γ_*a*_. These steps give

(10) $$\begin{array}{l} Q_{a}^{\left(0\right)}+Q_{a}^{\left(1\right)}\left(t\right)=S\left[V_{a}^{\left(0\right)}\right]+\rho_{a}V_{a}^{\left(1\right)}\left(t\right), \end{array}$$

(11) $$\begin{array}{l} {\hat{D}}_{\alpha }\left(t\right)\left[V_{a}^{\left(0\right)}+V_{a}^{\left(1\right)}\left(t\right)\right]=\underset{b}{\sum }\left[\nu_{ab}^{\left(0\right)}+\nu_{ab}^{\left(1\right)}\left(t\right)\right]\\ \;\times \left[\phi_{b}^{\left(0\right)}+\phi_{b}^{\left(1\right)}\left(t-\tau_{ab}\right)\right], \end{array}$$

(12) $$\begin{array}{l} {\hat{D}}_{a}\left(t\right)\left[\phi_{a}^{\left(0\right)}+\phi_{a}^{\left(1\right)}\left(t\right)\right]=Q_{a}^{\left(0\right)}+Q_{a}^{\left(1\right)}\left(t\right), \end{array}$$

(13) $$\begin{array}{l} \rho_{a}=\frac{dQ_{a}}{dV_{a}}{|}_{V_{a}=V_{a}^{\left(0\right)}}, \end{array}$$

(14) $$\begin{array}{l} {\hat{D}}_{a}\left(t\right)=\frac{1}{\gamma_{a}^{2}}\frac{\partial^{2}}{\partial t^{2}}+\frac{2}{\gamma_{a}}\frac{\partial }{\partial t}+1, \end{array}$$

To zeroth order Equations (10)–(12) yield

(15) $$\begin{array}{l} Q_{a}^{\left(0\right)}=S\left[V_{a}^{\left(0\right)}\right], \end{array}$$

(16) $$\begin{array}{l} V_{a}^{\left(0\right)}=\underset{b}{\sum }\nu_{ab}^{\left(0\right)}\phi_{b}^{\left(0\right)}, \end{array}$$

(17) $$\begin{array}{l} \phi_{a}^{\left(0\right)}=Q_{a}^{\left(0\right)}. \end{array}$$

Equations (15) and (17) can be used to eliminate the other variables in favor of the $V_{a}^{\left(0\right)}$, which yields the nonlinear steady-state equation (Robinson et al., 2002, 2004)

(18) $$\begin{array}{l} V_{a}^{\left(0\right)}=\underset{b}{\sum }\nu_{ab}^{\left(0\right)}S\left[V_{b}^{\left(0\right)}\right], \end{array}$$

where *b* runs over all populations, including *n*.

The first order terms in Equations (10)–(12) give

(19) $$\begin{array}{l} Q_{a}^{\left(1\right)}\left(t\right)=\rho_{a}V_{a}^{\left(1\right)}\left(t\right), \end{array}$$

(20) $$\begin{array}{l} {\hat{D}}_{\alpha }\left(t\right)V_{a}^{\left(1\right)}\left(t\right)=\underset{b}{\sum }\left[\nu_{ab}^{\left(0\right)}\phi_{b}^{\left(1\right)}\left(t-\tau_{ab}\right)+\nu_{ab}^{\left(1\right)}\left(t\right)\phi_{b}^{\left(0\right)}\right], \end{array}$$

(21) $$\begin{array}{l} {\hat{D}}_{a}\left(t\right)\phi_{a}^{\left(1\right)}\left(t\right)=Q_{a}^{\left(1\right)}\left(t\right), \end{array}$$

Operation with ${\hat{D}}_{\alpha }\left(t\right)$ on both sides of Equation (21), and substitution of (19) and (20) into the result, yields

(22) $$\begin{array}{l} {\hat{D}}_{\alpha }\left(t\right){\hat{D}}_{a}\left(t\right)\left[\phi_{a}^{\left(1\right)}\left(t\right)\right]=\rho_{a}{\hat{D}}_{\alpha }V_{a}^{\left(1\right)}\left(t\right), \end{array}$$

(23) $$\begin{array}{l} =\underset{b}{\sum }\left[G_{ab}^{\left(0\right)}\phi_{b}^{\left(1\right)}\left(t-\tau_{ab}\right)+G_{ab}^{\left(1\right)}\left(t\right)\phi_{b}^{\left(0\right)}\right], \end{array}$$

(24) $$\begin{array}{l} G_{ab}^{\left(0\right)}=\rho_{a}\nu_{ab}^{\left(0\right)}=\rho_{a}N_{ab}s_{ab}^{\left(0\right)}, \end{array}$$

(25) $$\begin{array}{l} G_{ab}^{\left(1\right)}\left(t\right)=\rho_{a}\nu_{ab}^{\left(1\right)}\left(t\right)=\rho_{a}N_{ab}s_{ab}^{\left(1\right)}\left(t\right)., \end{array}$$

The gain *G*_*ab*_(*t*) represents the differential change in output spike rate from neurons *a* per unit change in input spike rate from neurons *b*. The net gains of populations of neurons connected serially are denoted by *G*_*abc*_ = *G*_*ab*_*G*_*bc*_ and *G*_*abcd*_ = *G*_*ab*_*G*_*bc*_*G*_*cd*_.

#### 2.3.2. Modulation of Synaptic Gains

Many biophysical processes can modulate neuronal coupling strengths, and hence $s_{ab}^{\left(1\right)}$ in Equation (25), dependent on current or recent activity, including plasticity, long-term potentiation/depression, adaptation, facilitation, habituation, and sensitization (Koch, 1999; Rennie et al., 2000; Robinson and Roy, 2015; Babaie-Janvier and Robinson, 2019). We employ a general mathematical form of modulatory processes that can be applied to a broad range of specific mechanisms (Koch, 1999; Rennie et al., 1999; Robinson et al., 2002; Robinson and Roy, 2015), in which presynaptic neuronal activity locally modulates neuronal gains (dynamics driven by postsynaptic firing rate is postponed to future work, but can be treated in a similar way Rennie et al., 1999; Robinson and Roy, 2015), with

(26) $$\begin{array}{l} G_{ab}^{\left(1\right)}\left(t\right)=\left[g_{ab}F\left(t\right)+h_{ab}H\left(t\right)\right]⊗\phi_{b}^{\left(1\right)}\left(t\right), \end{array}$$

where the symbol ⊗ indicates a temporal convolution. Here *F*(*t*) describes the temporal dynamics of the fast gain modulation on timescales of up to a few hundred ms and *g*_*ab*_ is its strength, whereas *H*(*t*) is a slow adaptation process on timescales of several seconds, and *h*_*ab*_ is the corresponding strength.

Equation (26) assumes that the perturbations are small enough that a linear equation is a reasonable approximation. Furthermore, the modulation is assumed to be local in space, so the *g*_*ab*_ and *h*_*ab*_ are constant and the functional forms of *F*(*t*) and *H*(*t*) do not vary with position or time. For the temporal dependence of the modulation we use

(27) $$\begin{array}{l} F\left(t\right)=\eta \exp\left(-\eta t\right), \end{array}$$

(28) $$\begin{array}{l} H\left(t\right)=\mu \exp\left(-\mu t\right), \end{array}$$

when *t* ≥ 0 and *F*(*t*) = *H*(*t*) = 0 for *t* < 0 to enforce causality. The positive rate constants η and μ characterize the timescales of the modulatory processes and the forms (27) and (28) are normalized to unit integral over time. Previous work found that η = 25 s^−1^ is a reasonable choice (Rennie et al., 1999; Babaie-Janvier and Robinson, 2019), while we set μ = 0.65 s^−1^ because of the several-second timescales over which *S* response characteristics develop and decay.

#### 2.3.3. Transfer Functions

The transfer function is the ratio of the output of a system to its input in the linear regime. Either the Laplace or Fourier transform can be used to determine transfer functions; we use the former with the definitions

(29) $$\begin{array}{l} L\left[g\left(t\right)\right]\left(s\right)=\int_{0}^{\infty }g\left(t\right)e^{-st}dt. \end{array}$$

Application of Equation (29) to Equation (26) gives

(30) $$\begin{array}{l} {\hat{D}}_{b}\left(s\right)\left[\phi_{a}^{\left(0\right)}+\phi_{a}^{\left(1\right)}\left(s\right)\right]\\ \;=L\left(s\right)\underset{b}{\sum }\left[G_{ab}^{\left(0\right)}+\left\{g_{ab}F\left(s\right)+h_{ab}H\left(s\right)\right\}\phi_{b}^{\left(1\right)}\left(s\right)\right]\\ \;\times \left[\phi_{b}^{\left(0\right)}+\phi_{b}^{\left(1\right)}\left(s\right)\exp\left(-s\tau_{ab}\right)\right], \end{array}$$

(31) $$\begin{array}{l} {\hat{D}}_{b}\left(s\right)={\left(1+s/\gamma_{b}\right)}^{2}, \end{array}$$

(32) $$\begin{array}{l} L\left(s\right)={\left(1+s/\alpha \right)}^{-1}{\left(1+s/\beta \right)}^{-1}. \end{array}$$

Hence, to first order

(33) $$\begin{array}{l} {\hat{D}}_{b}\left(s\right)\phi_{a}^{\left(1\right)}\left(s\right)\\ \;=L\left(s\right)\underset{b}{\sum }\left[G_{ab}^{\left(0\right)}e^{-s\tau_{ab}}+\phi_{b}^{\left(0\right)}\left\{g_{ab}F\left(s\right)+h_{ab}H\left(s\right)\right\}\right]\phi_{b}^{\left(1\right)}\left(s\right), \end{array}$$

(34) $$\begin{array}{l} F\left(s\right)=\eta /\left(s+\eta \right), \end{array}$$

(35) $$\begin{array}{l} S\left(s\right)=\mu /\left(s+\mu \right). \end{array}$$

Equation (33) expresses two types of first order responses: the first term in the square brackets represents the part of response that would occur without change to the steady-state gains, while the second term is the response due to stimulus-induced gain changes acting on the steady-state activity.

Equation (33) represents a set of coupled algebraic equations that interrelate the $\phi_{a}^{\left(1\right)}$. It is straightforward to eliminate the other first order quantities to obtain the transfer function to excitatory cortical neurons from retinal signals that reach the thalamus (see Babaie-Janvier and Robinson, 2018 for detailed derivation), giving

(36) $$\begin{array}{l} T_{en}\left(s\right)=\frac{\phi_{e}^{\left(1\right)}\left(s\right)}{\phi_{n}^{\left(1\right)}\left(s\right)}, \end{array}$$

(37) $$\begin{array}{l} =\frac{A\left(s\right)}{q^{2}\left(s\right)r_{e}^{2}}, \end{array}$$

(38) $$\begin{array}{l} A\left(s\right)=\frac{X_{esn}}{\left(1-X_{ei}\right)\left(1-X_{srs}\right)}, \end{array}$$

(39) $$\begin{array}{l} q^{2}\left(s\right)r_{e}^{2}={\left(1+\frac{s}{\gamma_{e}}\right)}^{2}-\frac{1}{1-X_{ei}}\left[X_{ee}+\frac{X_{ese}+X_{esre}}{1-X_{srs}}\right], \end{array}$$

(40) $$\begin{array}{l} X_{ab}=L\left(s\right)\left[G_{ab}^{\left(0\right)}e^{-s\tau_{ab}}+\phi_{b}^{\left(0\right)}\left\{g_{ab}F\left(s\right)+h_{ab}H\left(s\right)\right\}\right]. \end{array}$$

### 2.4. Loop-Strength Representation

A useful and compact approximate representation of corticothalamic steady states and dynamics is via the normalized strengths of the corticocortical, corticothalamic, and intrathalamic loops in Figure 2. These are defined by (Robinson et al., 2002; Breakspear et al., 2006)

(41) $$\begin{array}{l} X=\frac{G_{ee}}{1-G_{ei}}, \end{array}$$

(42) $$\begin{array}{l} Y=\frac{G_{es}\left(G_{se}+G_{sr}G_{re}\right)}{1-G_{sr}G_{rs}}, \end{array}$$

(43) $$\begin{array}{l} Z=-\frac{\alpha \beta G_{sr}G_{rs}}{{\left(\alpha +\beta \right)}^{2}}, \end{array}$$

respectively. Originally defined with steady-state values of the *G*_*ab*_ on the right, Breakspear et al. (2006) later used instantaneous values of the time-varying *G*_*ab*_(*t*) to parameterize the orbits of dynamic states. Resonances in these loops are primarily responsible for the dominant frequencies in resting EEG and ERs (Kerr et al., 2008, 2011).

### 2.5. Control Systems Interpretation

Analysis and interpretation of the transfer function is greatly facilitated by approximating the quotient of exponential polynomials in (37) by a rational function of *s*. Decomposition into partial fractions then yields

(44) $$\begin{array}{l} T_{ab}\left(s\right)=\overset{n}{\underset{j=1}{\sum }}\frac{r_{j}}{s+p_{j}}; \end{array}$$

where the poles of the system are assumed to be distinct, with

(45) $$\begin{array}{l} p_{j}=\Gamma_{j}\pm i\Omega_{j}, \end{array}$$

where the damping rate is Γ_*j*_ and the frequency is Ω_*j*_; the residues *r*_*j*_ = *r* ± *i*Ω_*r*_ are

(46) $$\begin{array}{l} r_{j}=\underset{s\to -p_{j}}{\lim}\left(s+p_{j}\right)T_{ab}\left(s\right); \end{array}$$

and *n* is the number of the poles. Some poles are associated with heavily damped modes and can be neglected, thereby allowing *n* to be kept small. Indeed, a 6-pole approximation (*n* = 6) has been found to be accurate to within a root-mean-square (rms) fractional error of 0.02 for 0–150 Hz for the parameters in column 3 of Table 1 (Babaie-Janvier and Robinson, 2018). These partial fractions then are summed in pairs each of which dominates in slow/theta (*f* ≲ 5 Hz), alpha (5 Hz ≲ *f* ≲ 15 Hz), or beta (15 Hz ≲ *f*) frequency regimes, respectively. This gives

(47) $$\begin{array}{l} T_{bn}\left(s\right)\approx T_{bn}^{\ell }\left(s\right)+T_{bn}^{A}+T_{bn}^{B}\left(s\right), \end{array}$$

where *b* = *e, i, r, s* and $T_{bn}^{\ell }$, $T_{bn}^{A}$, and $T_{bn}^{B}$ are the sums over the pairs of poles that represent responses in the low, alpha, and beta frequency ranges, respectively. We denote the three corresponding partial transfer functions by $T_{ab}^{F}\left(s\right)$ for F=ℓ,A,B, with

(48) $$\begin{array}{l} T_{ab}^{F}\left(s\right)=\frac{r_{j}}{s+p_{j}}+\frac{r_{j+1}}{s+p_{j+1}}, \end{array}$$

where the poles *j* and *j* + 1 form a pair. Note that the poles and residues depend on F, *a*, and *b*, but we have not shown this explicitly to avoid unduly cumbersome notation.

Use of the partial fraction representation makes inversion of the Laplace transform straightforward. Two possibilities occur: either the two poles represent damped nonzero-frequency oscillations, and are complex conjugates, or they represent purely damped responses and are both real. In the oscillatory case, $p_{j+1}=p_{j}^{*}$ and $r_{j+1}=r_{j}^{*}$ so that the time-domain response is real. In this case, for a delta-function stimulus at *t* = 0,

(49) $$\begin{array}{l} T^{F}\left(t\right)=2|r_{j}|\exp\left(-\Gamma_{j}t\right)\cos\left(\Omega_{j}t-\psi \right), \end{array}$$

where we have written $r_{j}=|r_{j}|e^{i\psi }$. In the purely damped case, *r*_*j*_ and *r*_*j*+1_ are real but not equal and likewise for *p*_*j*_ and *p*_*j*+1_. This gives

(50) $$\begin{array}{l} T_{ab}^{F}\left(t\right)=r_{j}e^{-\Gamma_{j}t}+r_{j+1}e^{-\Gamma_{j+1}t} \end{array}$$

Equation (49), in particular, shows that analyses of ERs in terms of damped sinusoids (Freeman, 1975; Demiralp et al., 1998; Başar, 2012) are not just instances of compact phenomenology, but rest on the dynamics embodied in resonances of the transfer function. At a deeper level, each pair of poles can be interpreted as implementing a control systems data filter—specifically a PID (proportional-integral-derivative) filter—that predicts incoming signals based on signal value, rate of change, and integrated time history (Ogata and Yang, 1970; Babaie-Janvier and Robinson, 2018, 2019). Using this formulation, dynamic gain changes have been interpreted as improving prediction by implementing attention to salient features (Babaie-Janvier and Robinson, 2019).

### 2.6. Feature Map

The final generalization we require to the model is to incorporate different stimulus features, such as frequency. These are mapped to slightly different locations in the auditory cortex, which will adapt differently, so we need to distinguish them by a label σ. Here we assume that the ER measuring electrode responds equally to all relevant locations, although this is an assumption that could later be relaxed. In place of Equation (1) we write

(51) $$\begin{array}{l} \phi_{e}^{\left(1\right)}\left(\sigma ,t\right)=\int_{-\infty }^{t}T\left(t-t^{\prime }\right)\int w\left(\sigma -\sigma^{\prime }\right)\phi_{n}^{\left(1\right)}\left(\sigma^{\prime },t^{\prime }\right)d\sigma^{\prime }dt^{\prime }, \end{array}$$

where the weight function *w* quantifies the discriminability of stimuli; a suitable form is

(52) $$\begin{array}{l} w\left(\sigma -\sigma^{\prime }\right)=\exp\left[-\frac{{\left(\sigma -\sigma^{\prime }\right)}^{2}}{2{\left(\Delta \sigma \right)}^{2}}\right]. \end{array}$$

In Equation (51) we have assumed that *T* does not depend explicitly on σ, but such a dependence could easily be included. Aside from the issue of discriminability, and the inclusion of σ, the bulk of the above analysis is unchanged. However, the weight function *w* implies that stimuli σ′ within ~Δσ influence the dynamics at σ.

In the case of auditory stimuli, σ can be viewed as the frequency and a very short sinusoidal stimulus at *t*_0_ with frequency corresponding to σ_*A*_ has

(53) $$\begin{array}{l} \phi_{n}^{\left(1\right)}\left(\sigma^{\prime },t^{\prime }\right)\approx \delta \left(\sigma^{\prime }-\sigma_{A}\right)\delta \left(t^{\prime }-t_{0}\right). \end{array}$$

There is no sinusoidal time dependence in Equation (53) because the input pathway via the cochlea and superior colliculus translates each frequency to a point in the tonotopic map, without retaining its waveform. Using Equation (53), Equation (51) simplifies to

(54) $$\begin{array}{l} \phi_{e}^{\left(1\right)}\left(\sigma ,t\right)\approx w\left(\sigma -\sigma_{A}\right)T\left(t-t_{0}\right). \end{array}$$

If S and D stimuli are fully discriminable the only relevant values of *w*(σ − σ′) are 1 and 0; i.e., there is no cross-talk.

Note that, although we have assumed that σ is a scalar here, corresponding to stimulus frequency and the tonotopic auditory map, more generally it could be a vector of feature attributes, including frequency, interaural delay, intensity, and other quantities, with different sensoricortical maps (Herdener et al., 2013).

## 3. Results

We are now in a position to analyze the different responses to frequent and rare stimuli, which will ultimately evoke *S* and *D* responses, respectively, in a long sequence. In this section we assume that these two stimulus types are fully discriminable so we do not need to include the parameter Δσ of section 2.5 and simply denote the frequent and rare stimuli by σ=S and σ=D, respectively. We optionally denote the *n*th consecutive stimulus of a given type by the subscript *n*; i.e., $S_{n}$ describes the *n*th consecutive S stimulus and $D_{n}$ denotes the *n*th consecutive D stimulus. We write the corresponding system responses, which can differ between presentations of the same stimulus type, due to adaptation, as *S*_*n*_(*t*) and *D*_*n*_(*t*) but omit the argument *t* when referring to the entire response. Standard responses rapidly approach a limiting form *S*_∞_(*t*) after a few (typically about *n* = 5) presentations of S, with little change thereafter (Cowan, 1984; Winkler et al., 1993; Loveless et al., 1996). The MMN between the *n*th *D* response and the *m*th *S* response, for example, is defined to be the difference

(55) $$\begin{array}{l} \text{MMN}\left(D_{n},S_{m},t\right)=D_{n}\left(t\right)-S_{m}\left(t\right), \end{array}$$

with analogous definitions for other pairs of responses. Most commonly the MMN is defined to be MMN(*D*_1_, *S*_∞_, *t*) the version obtained by subtracting the limiting form *S*_∞_(*t*) of the standard response from *D*_1_(*t*). Note that the first responses to both stimuli are identical because both are novel: *S*_1_(*t*) = *D*_1_(*t*).

In this section we first explain the formulation of differential adaptation to S and D stimuli. We then calibrate the parameters *g*_*ab*_ and *h*_*ab*_ of the model by matching their predictions for $\phi_{e}^{\left(1\right)}\left(t\right)$ to typical oddball data before applying the results to a variety of other stimulus sequences in later subsections. These results allow exploration of which MMN effects can be accounted for by adaptation in the primary auditory cortex and the medial geniculate nucleus of the thalamus.

### 3.1. Contribution of Adaptation to Responses

**Figure 3:**
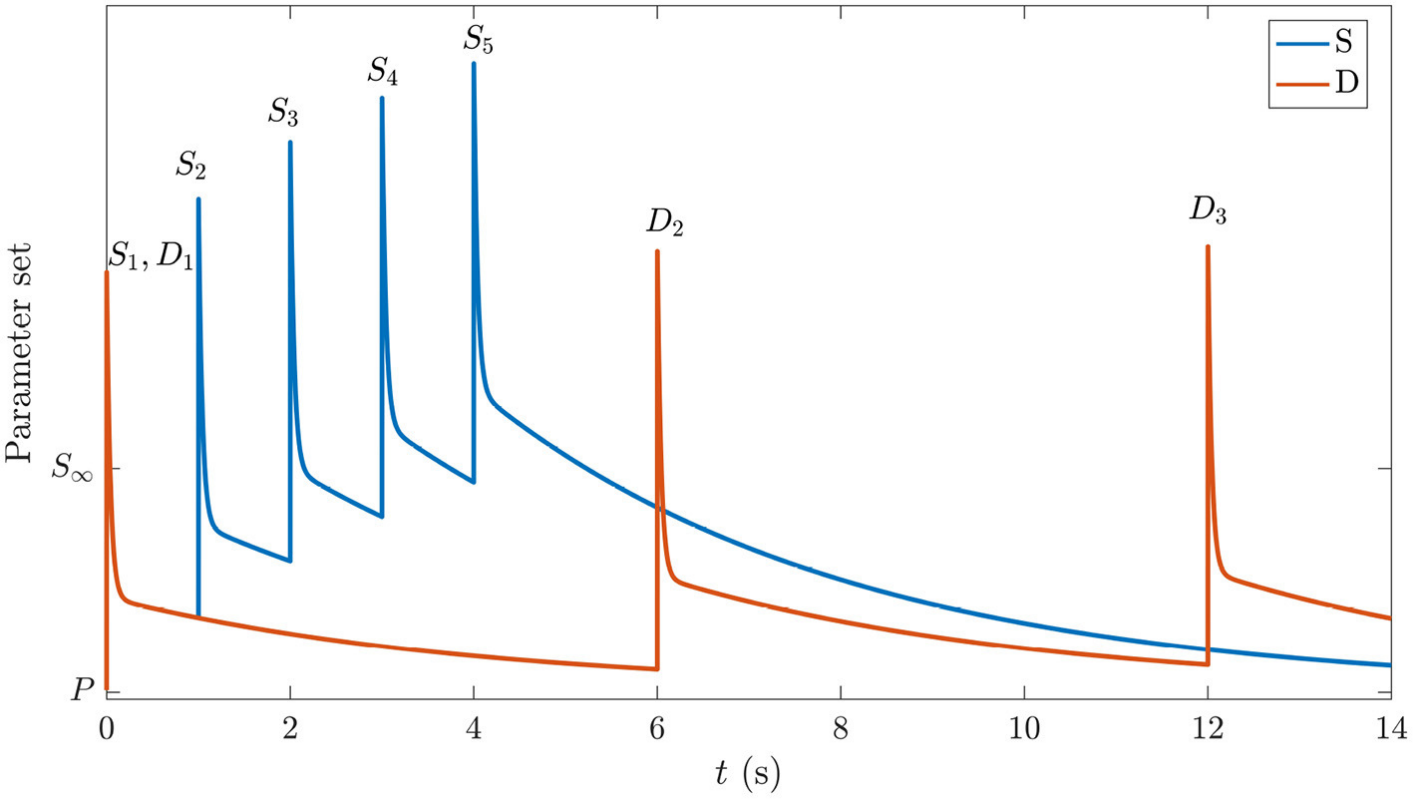
Schematic of gain responses of the pre-stimulation (P) state and the trajectories followed by the different parts of the corticothalamic system that process the S and D stimuli. The vertical axis schematically represents the response of the set of system gains, with starting point P and the asymptotic value in the *S*_∞_ response labeled. The horizontal axis indicates time and is labeled with stimulus types and numbers. The blue curve corresponds to stimuli $S_{1},\ldots ,S_{5}$ and the orange curve corresponds to $D_{1},\ldots ,D_{3}$.

The central idea used here is that the system initially occupies the same prestimulation state as the one corresponding to background EEG in the period before any stimuli have been presented. We label this P in the schematic space of gains in Figure 3. In the absence of adaptation, each stimulus causes transient gain changes due to the term *F*(*t*) in Equation (26). However, because the time constant of *H*(*t*) is roughly 40 times larger, resulting changes due to that process may not have fully relaxed by the time the next stimulus arrives. Hence, the system will be pushed to the location corresponding to *S*_∞_ by a long series of S stimuli (blue curve in Figure 3), eventually oscillating around a point where

(56) $$\begin{array}{l} \Delta G_{ab}\sim g_{ab}{\langle \phi_{b}^{\left(1\right)}\left(t\right)\rangle }_{\eta }+h_{ab}{\langle \phi_{b}^{\left(1\right)}\left(t\right)\rangle }_{\mu }, \end{array}$$

where the angle brackets denote an average over the most recent time interval of order 1/η or 1/μ, as indicated by the subscript; these averages are nonzero in general because incoming delta-function stimuli have a nonzero mean. The first average decays within tens of ms, and can usually be neglected by the time the next stimulus arrives, but the second can be significant in a train of S stimuli. In contrast, in the case of D stimuli, which come more rarely, the system gains will have time to relax almost to P in the interim (orange curve in Figure 3). Hence, we argue that deviant responses occur from near-P conditions, whereas standard responses occur from a location in parameter space that shifts gradually toward the parameters of *S*_∞_ over several stimuli. We see from Figure 3 that the *S* gain response doesn't get a chance to relax back to P due to the shortly-spaced stimuli whereas the *D* gain response is triggered relatively rarely and decays back almost to P between stimuli. Both responses to the first stimulus are the same.

One key point above is that each ER starts from the relevant time-evolving gains after the previous stimulus, or else all responses would simply be added with the same functional form. It might be objected that this amounts to retention of a second order term in the perturbation expansion and that we should therefore retain all second order terms. However, although this point of view is formally correct, it is not necessary to retain the other second-order terms because the long time constant of *H*(*t*) effectively “promotes” its formally first-order effects by integrating over several seconds to produce changes that are comparable with zeroth-order terms. It is only after times ≳5 − 10 s without stimuli that these changes decay and the system again approaches the state P. Indeed, Equations (27) and (28) show that terms arising from *H*(*t*) are of order exp[(η − μ)*t*] larger than those arising from *F*(*t*) a time *t* after a stimulus; this can be a very large factor and these terms cannot generally be neglected relative to zeroth-order gains.

### 3.2. Parameter Calibration

A comparison of the present pre-stimulus gain parameters $G_{ab}^{\left(0\right)}$ corresponding to the baseline EEG state (P) with those used in previous work that reproduced Standard ERs with static-gains (Kerr et al., 2008) and with modified gains (Babaie-Janvier and Robinson, 2020) can be done by comparing the third column of Table 1 with the fourth and fifth columns, respectively. The present P parameters are identical to those of Babaie-Janvier and Robinson (2020) except for slight changes in $G_{ee}^{\left(0\right)}$ and $G_{re}^{\left(0\right)}$, and are mostly larger than those used by Kerr et al. (2008) except for *G*_*rs*_ and *G*_*re*_. Of course, we do not expect exact correspondence because the previous studies did not include slow adaptation via *H*(*t*).

**Figure 4:**
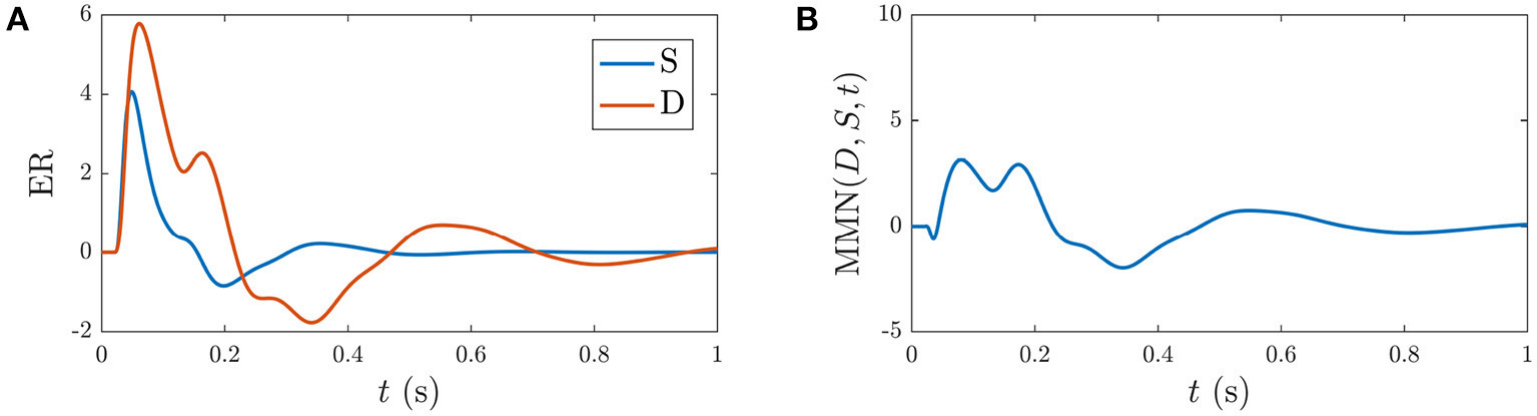
Static-gain ERs calibrated to closely approximate typical *S* and *D* responses from Kerr et al. (2008), Kerr et al. (2011), and Babaie-Janvier and Robinson (2019), as indicated in the legend, which were used as benchmarks for the *S*_∞_ and *D* responses (outlined in section 3.2). **(A)** Benchmark *S* and *D* curves used to derive gain modulations in Table 2. **(B)** Corresponding MMN(*D, S, t*).

Based on previous NFT analysis of standard and deviant responses (Kerr et al., 2008, 2011) and recent work which analyzed the role the different gains play in determining ER features (Babaie-Janvier and Robinson, 2019), we derived static-gain ERs above by adjusting the $G_{ab}^{\left(0\right)}$ in the transfer function component of Equation (40) and setting *g*_*ab*_ = 0 and *h*_*ab*_ = 0. These served as benchmarks for the *S*_∞_ and *D* responses shown in Figure 4. The key difference between these two curves is the relative increase of the corticothalamic loop gains $G_{es}^{\left(0\right)}$ and $G_{se}^{\left(0\right)}$ as well as the top-down pathway $G_{re}^{\left(0\right)}$ for *D* relative to *S*, resulting in the presence of the N2 and P2 features in *D*. It is worth noting that a variety of standard and deviant responses are found in the literature, due in part to slightly varied experimental conditions, and that our present aim is not to reproduce a particular set of response curves exactly, but rather to incorporate common features such as the standard response being reduced in amplitude and lacking the widely established N2 deflection that is often seen and interpreted as contributing to the MMN (Näätänen et al., 1978, 1989a; Sams et al., 1984).

Building on findings and recent estimates of local feedback modulation in *g*_*ab*_ that give rise to ERs (Babaie-Janvier and Robinson, 2019), we calibrate the model parameters *g*_*ab*_ and *h*_*ab*_ by minimizing the error between the benchmark curves and the model-calculated curves such that the resulting activity resembled the *S* benchmark upon a number of closely-spaced, consecutive stimulation, and resembled the *D* benchmark upon less-frequent stimulation. These calibrated parameters are shown in Table 2 and discussed with respect to their contributions to the activity responses in the next section.

**Table 2:** Characteristic fast and slow gain response parameters and their percentage change relative to the static baseline parameters $G_{ab}^{\left(0\right)}$ (which are dimensionless).

**Parameter**	**Value**	**$\Delta |G_{ab}^{\left(0\right)}|$ (%)**
**FAST CHANGE CONTRIBUTION**		
ηg_*ee*_	−0.1157	−2
ηg_*ei*_	0.7684	9
ηg_*es*_	0.7419	44
ηg_*se*_	0.1047	4
ηg_*sr*_	−0.1390	7
ηg_*rs*_	0.1149	60
ηg_*re*_	0.2822	22
**ADAPTATION CONTRIBUTION**		
μh_*ee*_	0.4390	7
μh_*ei*_	− 1.1180	14
μh_*es*_	−0.0890	−5
μh_*se*_	−0.3299	−13
μh_*sr*_	0.0053	0.3
μh_*rs*_	0.0018	1
μh_*re*_	−0.0969	−7

*In each case, the parameter is multiplied by its inverse timescale to obtain a characteristic contribution, as seen from Equations (27) and (28)*.

Here we analyze the local feedback strengths *g*_*ab*_ and *h*_*ab*_, presented in Table 2, that give rise to successive *S*_*n*_ and *D*_*n*_ responses. The slow adaptation contributions, parameterized by the *h*_*ab*_, determine the gradual evolution of the baseline of responses due to a series of stimuli over several seconds, while fast gain changes parameterized by the *g*_*ab*_ primarily determine the shape of the responses on the few-hundred ms scale, with differences between *S*_∞_ and *D*_1_ resulting from their different starting points.

**Figure 5:**
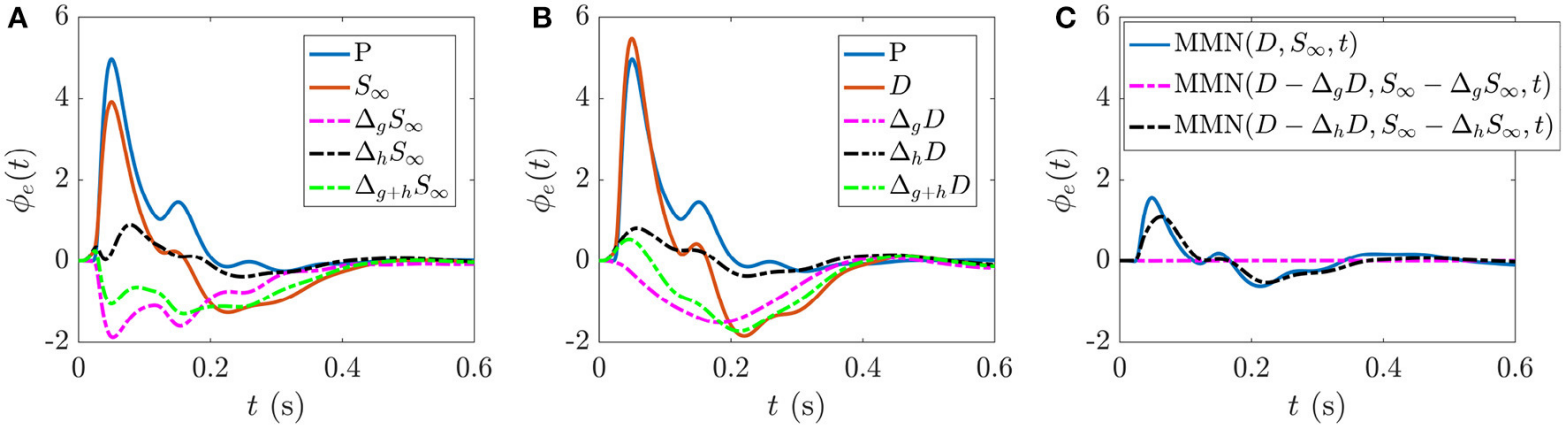
ERs and gain contributions corresponding to different responses. **(A)** Static gain baseline ER starting from P, fast gain modulation Δ_*g*_*S*_∞_ and adaptation Δ_*h*_*S*_∞_ contributions which, when added together (Δ_*g*+*h*_*S*_∞_) and combined with the baseline ER give the *S*_∞_ response. **(B)** Static gain baseline ER starting from P, fast gain modulation Δ_*g*_*D*, and adaptation Δ_*h*_*D* contributions which, when added together (Δ_*g*+*h*_*D*) and combined with the baseline ER give the *D* response. **(C)** MMN(*D, S*_∞_, *t*) and the corresponding isolated fast gain modulation (magenta broken line) and adaptation (black broken line) contributions.

The present work allows us to distinguish the parts of *S*_∞_ and *D* responses that are specifically due to fast gain modulations and slower adaptation. Figure 5A shows the static gain baseline ER (starting at P) along with the fast and slow gain modulation contributions to the *S* response,

(57) $$\begin{array}{l} \Delta_{g}S_{\infty }\left(t\right)=S_{\infty }\left(t\right)-S_{\infty }\left(t\right){|}_{h_{ab}=0}, \end{array}$$

(58) $$\begin{array}{l} \Delta_{h}S_{\infty }\left(t\right)=S_{\infty }\left(t\right)-S_{\infty }\left(t\right){|}_{g_{ab}=0}, \end{array}$$

which are the differences between the total *S*_∞_ response and the *S*_∞_ responses due to setting all *h*_*ab*_ = 0 and *g*_*ab*_ = 0, respectively. The combined effect of these individual processes (green broken line) is calculated according to

(59) $$\begin{array}{l} \Delta_{g+h}S_{\infty }\left(t\right)=S_{\infty }\left(t\right)-S_{\infty }\left(t\right){|}_{g_{ab}=0,h_{ab}=0}, \end{array}$$

and combines with the baseline ER (blue line) to give the *S*_∞_ response (orange line). We note that fast gain modulations act to decrease the N1 and N2 deflections in the baseline ER, whereas the adaptation contribution is smaller in magnitude and predominantly increases the N1 deflection. The overall local gain modulation contribution decreases the N1 and N2 deflections in the baseline ER and produces a deflection at ≈200–300 ms, giving the *S*_∞_ response.

Analogously, Figure 5B shows the static gain baseline ER (starting at P) alongside the fast gain and adaptation contributions Δ_*g*_*D* and Δ_*h*_*D* which sum with the baseline ER to give the *D* response. We note that fast gain modulations exhibit a P2 deflection at a slightly earlier latency compared to the D response, whereas the adaptation contribution exhibits small N1 and N2 deflections. The overall local gain modulation contribution thus slightly increases the N1 amplitude, reduces the N2 amplitude, and introduces a large P2 deflection at ≈200–300 ms in the baseline ER state to give rise to the D response.

Figure 5C explores how these distinct gain contributions affect the MMN. Calculating MMN(*D* − Δ_*g*_*D, S*_∞_ − Δ_*g*_*S*_∞_, *t*) isolates the part of the MMN that is caused by fast gain modulations (broken magenta line) and calculating MMN(*D* − Δ_*h*_*D, S*_∞_ − Δ_*h*_*S*_∞_, *t*) exposes the part of the MMN that is caused by adaptation (broken black line). As expected, without adaptation there is negligible distinction between the two responses at the short timescales shown, so the MMN is zero. The difference between the MMN(*D, S*_∞_, *t*) curve (blue line) and the adaptation contribution (black broken line) reveals how turning on the fast gain modulations affects the resultant MMN shape; the fast gain modulations act to slightly increase the amplitude of N1 and N2 features of the MMN.

As can be seen in Table 2, the dominant percentage changes in fast gain dynamics occur in the bottom-up pathways *g*_*es*_ and *g*_*rs*_, and to a lesser extent in the top-down pathway *g*_*re*_ whereas the dominant percentage changes in slow adaptation occur in the cortical and top-down pathways *h*_*ei*_ and *h*_*se*_, and to a lesser extent in the cortical, bottom-up, and top-down pathways *h*_*ee*_, *h*_*es*_, and *h*_*re*_. These findings suggest that cortical and top-down pathways play enhanced roles in adaptation to produce *S* responses.

These results generalize recent work that only considered fast change contributions to local feedback modulations *g*_*ab*_ (Babaie-Janvier and Robinson, 2019). In agreement with that work, we find a decrease of the inhibitory cortical and intrathalamic gains *g*_*ee*_ and *g*_*sr*_ and an increase in top-down corticothalamic gain *g*_*re*_. Although, in contrast, we found increases in the cortical inhibitory, top-down corticothalamic, and bottom-up thalamocortical gains *g*_*ei*_, *g*_*se*_, and *g*_*es*_ rather than decreases.

### 3.3. Development and Decay of Responses

**Figure 6:**
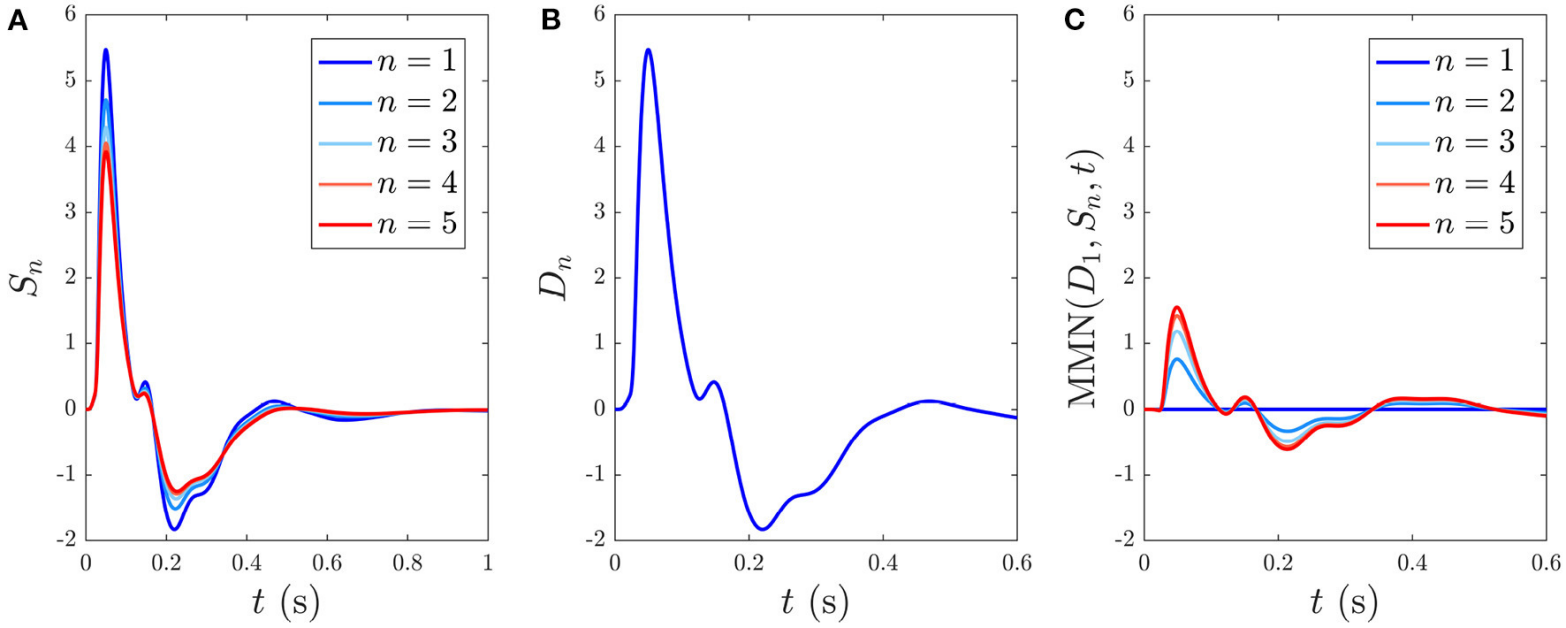
Development of responses *S*_∞_(*t*) and *D*(*t*) over multiple stimuli due to their different ISIs, with stimulus numbers indicated in the legends. **(A)** *S*_*n*_(*t*) for *n* = 1, …, 5 with a 1 s ISI. **(B)** *D*_*n*_(*t*) for *n* = 1, which is indistinguishable on this scale from cases with *n* > 1 with a 6 s ISI. **(C)** The MMN(*D*_1_, *S*_*n*_, *t*) corresponding to the sequential *S*_*n*_ responses from **(A)**.

Here we analyze the development and decay of the *S*_∞_ and *D* responses during long trains that are distinguishable by their different ISIs. Figure 6A shows the evolution of *S*_*n*_(*t*) toward the *S*_∞_(*t*) response for *n* = 1, …5 with 1 s ISI. As expected, the initial response to the first standard stimulus $S_{1}$ is a deviant response, *S*_1_ = *D*_1_, which does not exhibit as strong an N2 deflection as the benchmark in Figure 4A and the latency of the N2 peak is slightly earlier (≈ 10 ms) than the benchmark due to the effects of the *g*_*ab*_ and *h*_*ab*_. Importantly, *D*_1_ contains the N1 and P2 deflections, as seen in experiments (Garrido et al., 2009b). The gradual adaptation of *S*_*n*_ with *n* is also seen; little further change is found to occur after *n* = 5. The response *S*_5_(*t*) ≈ *S*_∞_(*t*) shows a reduction in N1 and N2 amplitudes relative to *D*_1_, which has also been experimentally observed (Sams et al., 1984; Cowan et al., 1993; Garrido et al., 2009b).

When the ISI is increased to the typical value for D stimuli in auditory oddball paradigms almost identical *D*_*n*_(*t*) responses emerge, independent of *n*, as seen in Figure 6B, which shows the system response to five stimuli with 6 s ISI. This occurs because there is sufficient time for the parameters to relax very nearly to the prestimulation state P between stimuli. We thus refer to deviant responses as *D*(*t*) without subscript from now on unless otherwise specified.

The development of MMN(*D*_1_, *S*_*n*_, *t*) vs. *n*, defined in Equation (55), is seen in Figure 6C. We see that the MMN is zero at *n* = 1 and grows with *n* as adaptation occurs in response to successive S stimuli. This MMN is positive in the 20–180 ms range, in agreement with experimental findings (Cowan et al., 1993; Garrido et al., 2007, 2009b; Näätänen et al., 2007).

**Figure 7:**
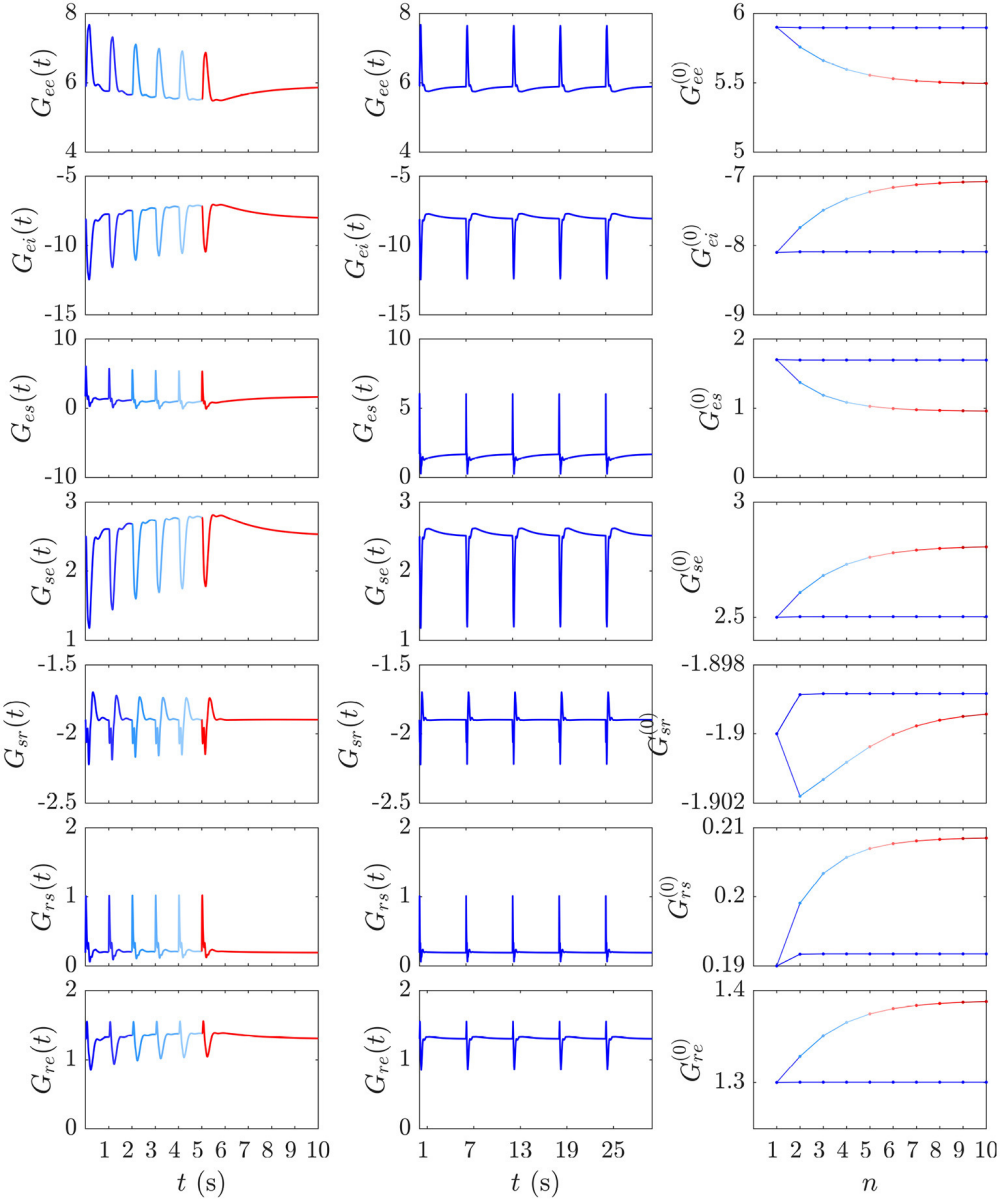
Evolution of the instantaneous *G*_*ab*_(*t*) and prestimulus $G_{ab}^{\left(0\right)}$ gains corresponding to the two cases from Figures 6A,B. The first and second columns show the gain evolution *G*_*ab*_(*t*) during the development of the *S*_∞_ response (blue to red) and the successive D responses (blue), respectively. The third column depicts the corresponding pre-stimulus gains $G_{ab}^{\left(0\right)}$ immediately prior to the successive S and D stimuli vs. stimulus number *n*.

The gain dynamics corresponding to the stimulus sequences in Figures 6A,B, followed by a stimulus-free interval, are illustrated in the first two columns of Figure 7, which further underlines how the development of *S*_∞_(*t*) involves the gains approaching a new baseline as adaptation occurs. In the stimulus-free interval after *t* = 5 s the gains decay back to their *P*-values on a timescale of roughly 5 s.

Our expectation that *D* responses should occur from near-P conditions while *S*_∞_ responses occur from a shifted starting point in parameter space is confirmed by examining prestimulus gains at the moment of each successive stimulus in the S and D streams above. Figure 7 (third column) shows these gains just before each stimulus vs. stimulus number for the S and D streams; it is evident that the S stream pushes the system further from P while the D stream allows it to relax back to near P at the time of the next such stimulus.

**Figure 8:**
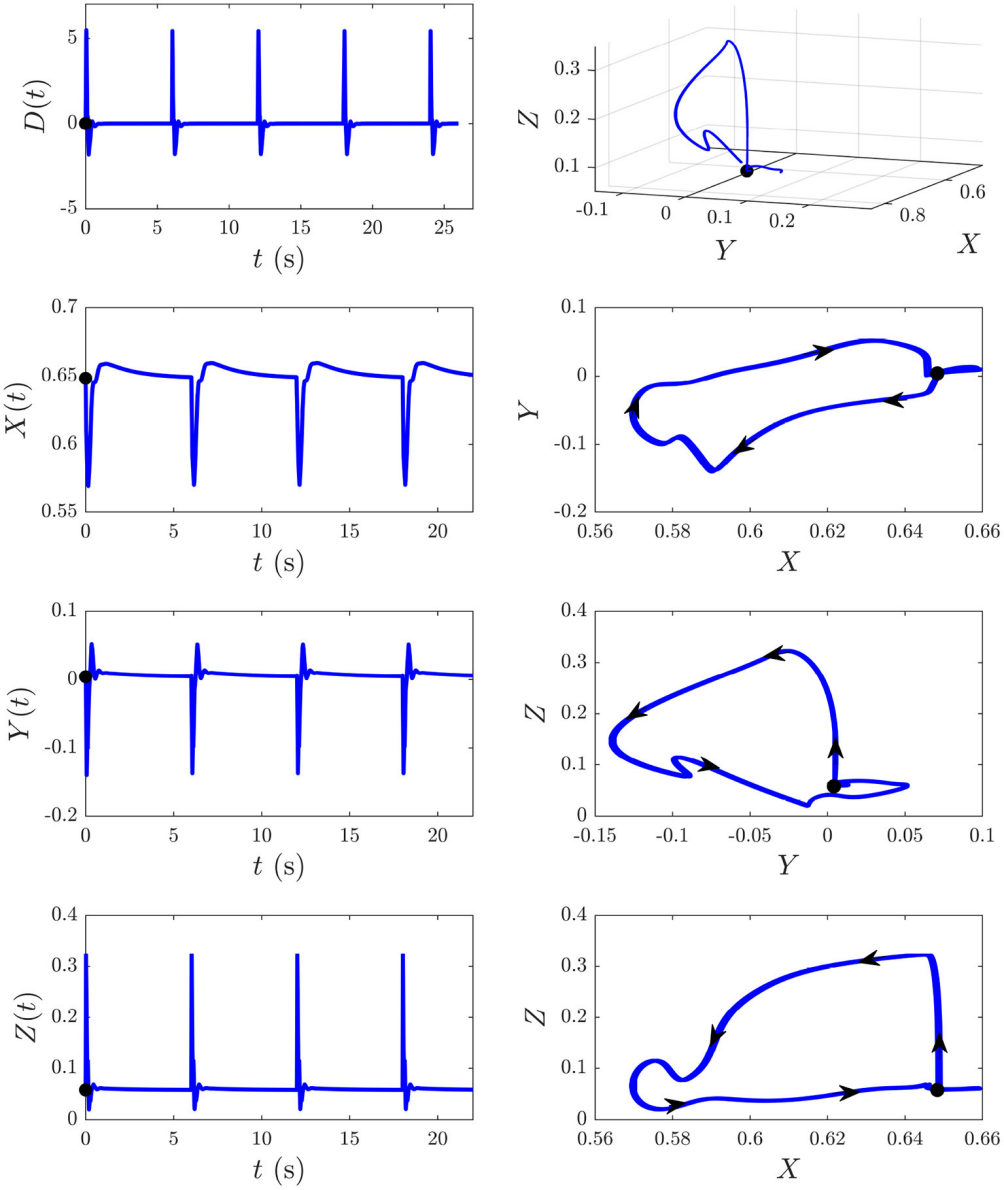
The *D* responses from Figure 6B and their associated trajectories in *XYZ* space (*X*, *Y*, and *Z* are intracortical, corticothalamic, and intrathalamic loop strengths, respectively). The black dot indicates the start point *t* = 0. First column shows the sequence of evoked responses at 6 s ISI and corresponding *X*(*t*), *Y*(*t*), and *Z*(*t*). Second column shows this trajectory in *XYZ* space and in the *XY*, *YZ*, and *XZ* planes, where the arrows indicate the direction of motion.

We now explore the *S* and *D* responses from Figure 6 in *XYZ* space, illuminating how cortical, corticothalamic, and intrathalamic feedback loops contribute to such dynamics. We first analyze the *D* response from Figure 6B and then explore the development of *S*_∞_(*t*) from Figure 6A. Figure 8 shows the sequence of evoked responses and their corresponding *X*(*t*), *Y*(*t*), and *Z*(*t*) (first column) alongside the trajectory they traverse in *XYZ* space, and the *XY*, *YZ*, and *XZ* planes (second column). A video of this activity is provided in Supplementary Video 1. As can be most easily seen in the video but also evident in this figure, each *D* response follows almost the same path in *XYZ* space. Each loop of the trajectory is characterized by an initial sharp increase of *Z* from ≈ 0.05 to 0.3 until *t* ≈ 25 ms post-stimulus, followed by a decrease of all coordinates. Then *Y* starts to increase at *t* ≈ 70 ms while *X* and *Z* continue to decrease until *t* ≈ 100 ms, at which point there is a short-lived rise in *Z*. Then *X* increases substantially, taking the system back to its starting point; *Y* also increases during this phase, becoming briefly positive before peaking at *t* ≈ 380 ms and returning to the starting value of *Y* ≈ 0. Around *t* = 600 ms the trajectory displays a small excursion from near its starting point as *X* travels further in the positive *X* direction (from *X* ≈ 0.65 − 0.66, most evident in the *XY* and *XZ* plane plots) before decaying back to its starting point by *t* ≈ 4 s. This is because of the relatively long-lasting shifts in *G*_*ee*_(*t*) and *G*_*ei*_(*t*) [which determine *X*(*t*)] in the second column of Figure 7. This suggests that during ERs, shifts in intracortical feedback strengths take longer to return to baseline than corticothalamic and intrathalamic ones.

**Figure 9:**
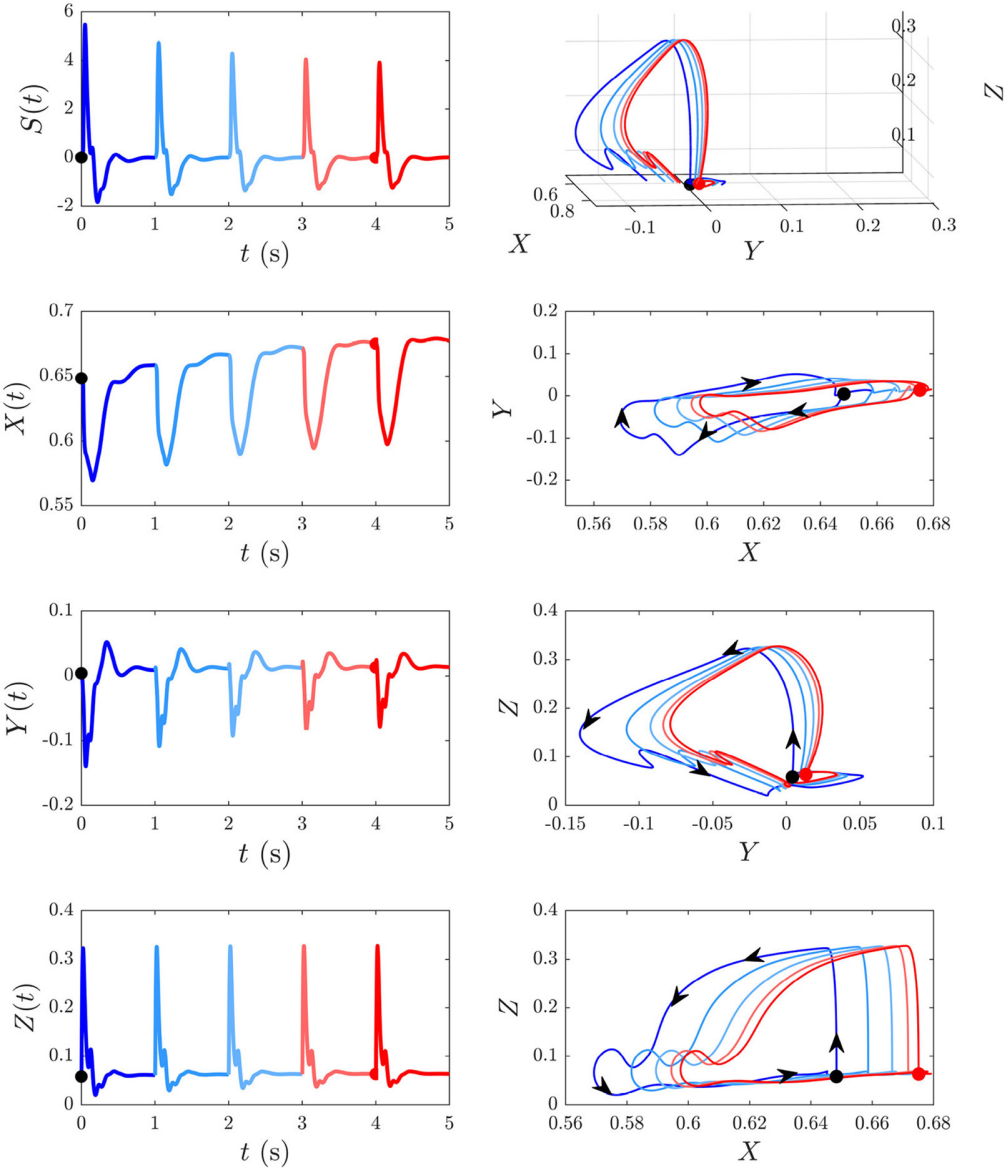
Development of the *S*_∞_ response from Figure 6B and its associated trajectories in *XYZ* space. The black dot indicates the start point at baseline P and the red dot indicates the start point *S*_∞_. First column shows the evoked response from Figure 6, and the corresponding *X*(*t*), *Y*(*t*), and *Z*(*t*) curves (*X*, *Y*, and *Z* are intracortical, corticothalamic, and intrathalamic loop strengths, respectively). Second column shows this trajectory in *XYZ* space, and in the *XY*, *YZ*, and *XZ* planes where the arrows indicate the direction of motion.

We now examine the development of the *S*_∞_ response from Figure 6A. As can be seen from the third column of Figure 7, the development of this response is accompanied by a 7% decrease in *G*_*ee*_, 13% decrease in |*G*_*ei*_|, 44% decrease in *G*_*es*_, 12% increase in *G*_*se*_, ≈ 0% change in *G*_*sr*_, 10% increase in *G*_*rs*_, and a 7% increase in *G*_*re*_. Figure 9 shows the evoked response corresponding to the development of *S*_∞_(*t*), the corresponding *X*(*t*), *Y*(*t*), and *Z*(*t*) (first column) alongside the trajectory it traverses in *XYZ* space, and the *XY*, *YZ*, and *XZ* planes (second column), with arrows indicating the direction of motion. A video of this activity is provided in Supplementary Video 2. The first thing to note is that, as expected, the first orbit (deep blue) is identical to *D*(*t*) from Figure 8. As adaptation occurs, the starting point for activity moves roughly in the positive *X* direction, as seen in Figure 9, with smaller shifts in *Y* and *Z*. The dominant shift in *X* is a consequence of the abovementioned fact that *X* takes the longest to decay back to its baseline value; i.e., the starting points for successive stimuli shift further along the *X* axis. It also implies that the dominant changes in brain dynamics occurring during adaptation to S stimuli involve increased intracortical feedback followed by increased corticothalamic and intrathalamic feedback, respectively.

### 3.4. Sequences of Stimuli

Here we move on from investigating the *S* and *D* responses individually and analyze a variety of different sequences of S and D stimuli that have been implemented experimentally. Recall that in the present analysis S and D stimuli are fully distinguishable and there is no cross-talk in Equation (54) so we can model each response separately. After several consecutive identical stimuli are interrupted by an occurrence of the other type of stimulus, *n* is reset to 1 here, so a given *n* can correspond to different situations, depending on what has occurred in the previous 5–10 s. We also introduce the following additional notation for brevity: a stream of *m* consecutive S stimuli is written as (mS). It is important to note that the responses to individual stimuli in (mS) are not identical and depend on the history. For example, in the sequence $\left(mS\right)D_{1}\left(mS\right)$, the first set of *S* responses are not identical to the corresponding members of the second set because the latter start from a more adapted corticothalamic region than the first. (The occurrence of the single $D_{1}$ stimulus does not give enough time for the next S stimulus to start from the same baseline as the very first stimulus in the sequence, so the residual adaptation arising from the first group of S stimuli is still significant when the second set commences.) The present analysis provides a firm, physiologically-based footing from which to analyze to what extent adaptation plays a role in the many different features of the MMN, and in which case higher-order processes are involved. Because digitized experimental data are not available from the literature we cannot fully calibrate our model to individuals, so the comparisons presented are necessarily semiquantitative.

#### 3.4.1. Sequence of Standards With Occasional Single Deviants

We first model an early study (Sams et al., 1984) which presented standard tones (1,000 Hz) 90% of the time and deviant tones (1,250 Hz) 10% of the time in random order and calculated the MMN corresponding to the first deviant tone after at least four standard tones. Each block contained 500 tones with 1 s ISI and the ERs to standard and deviant tones were separately averaged. We simulate a block by generating sequences of random S and D stimuli with the above probability distribution, only considering cases where a D stimulus immediately follows at least four S stimuli.

**Figure 10:**
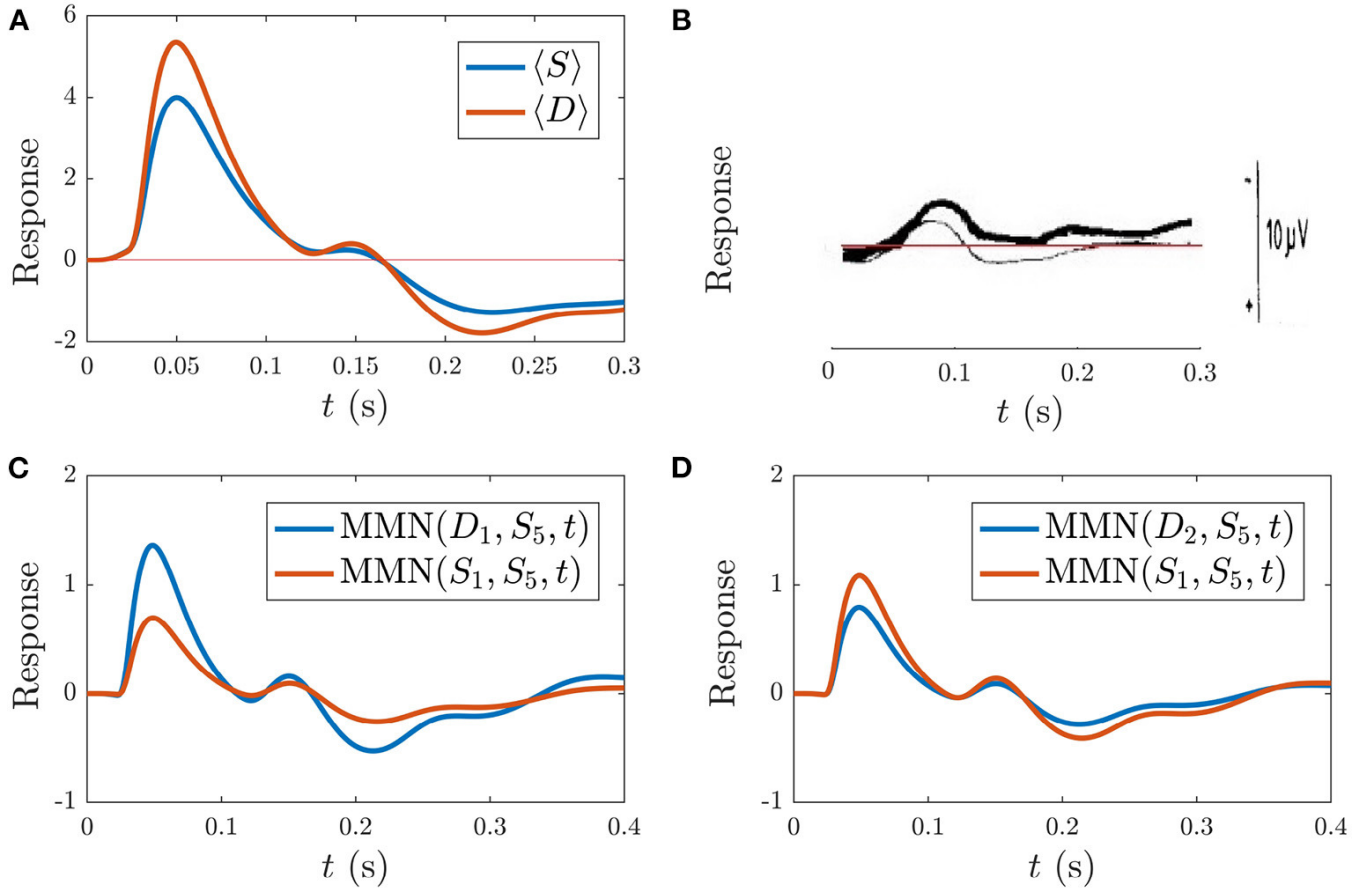
Simulations vs. experiment (Sams et al., 1984) for the sequences discussed in section 3.4.1. **(A)** Model *S* and *D* (see legend), each averaged over all presentations during the typical sequence of standards with occasional deviants. **(B)** Corresponding experimental findings from this same sequence, adapted from Sams et al. (1984), where *D* is indicated with the thin line and *D* is indicated by the bold line. The experimental scale bar at right is 10 μV in length. **(C)** Model findings for the MMN(*D*_1_, *S*_5_, *t*) and MMN(*S*_1_, *S*_5_, *t*) (see legend) corresponding to the sequence $\left(4S\right)S_{5}D_{1}S_{1}$. **(D)** Model findings for the MMN(*D*_2_, *S*_5_, *t*) and MMN(*S*_1_, *S*_5_, *t*) corresponding to the sequence $\left(4S\right)S_{5}D_{1}D_{2}S_{1}$.

Figure 10 shows the model simulation comparison to the experimental ER findings. Figures 10A,B display the resultant model averaged *S* and *D* responses over all instances of each stimulus as well as the experimental ERs from Sams et al. (1984), respectively. The model *S* and *D* responses reproduce the main features of the experimental responses: the *S* response displays prominent N1 and P2 deflections with similar latencies to the experimental *S* response, while *D* has a larger N1 peak than *S* and an N2 peak of lower amplitude than N1, which is also the case experimentally. The model findings for *D* differ from experiment in there being a prominent P2 deflection at *t* > 170 ms which isn't seen in the experimental response. This difference could signify the presence of higher-level feedbacks, or it may merely indicate that we have not adjusted our parameters to optimize the fit to this specific experiment. The fact that we see reasonable agreement between model simulations and experiment without further adjustments is evidence that adaptation plays a significant role in the development of the MMN. In future, fits to high-quality data for multiple experiments done on the same subjects should resolve this issue. Note that the timings of deflections in the model responses change depending on model parameters, unlike the fixed timings of traditional ER components.

In addition to calculating the MMN corresponding to the first D after four or more S as defined above, the experimental study tested whether an S stimulus directly following the D stimulus also caused a MMN with respect to the S preceding the D. Specifically, they implemented the following sequence of stimuli: $\left(4S\right)S_{5}D_{1}S_{1}$ and calculated MMN(*S*_1_, *S*_5_, *t*). They found that $S_{1}$ did indeed yield a nonzero MMN(*S*_1_, *S*_5_, *t*), albeit smaller in amplitude than the MMN(*D*_1_, *S*_5_, *t*) (Sams et al., 1984). Simulation of this sequence yields results in agreement with these findings. Figure 10C shows MMN(*S*_1_, *S*_5_, *t*) alongside the MMN(*D*_1_, *S*_5_, *t*), revealing that the latter is larger in amplitude, in agreement with experiment.

The extent to which our model reproduces the above experiments sheds light on the role adaptation plays in these particular scenarios. Historically, the experimental observation of the MMN(*S*_1_, *S*_5_, *t*) was interpreted via an argument that each stimulus is associated with its own “neuronal model” such that $D_{1}$ not only causes a mismatch process relative to the neuronal model corresponding to S stimuli, but also initiates a neuronal model of its own (Sams et al., 1984). The present findings suggest that adaptation is able to account for the MMN(*S*_1_, *S*_5_, *t*) being smaller in amplitude than MMN(*D*_1_, *S*_5_, *t*) in this experiment, without needing to invoke such higher-order neuronal models or representations.

#### 3.4.2. Sequence of Standards With Occasional Double Deviants

The authors from the study examined in the previous section also explored sequences of the form: $\left(4S\right)S_{5}D_{1}D_{2}S_{1}$, whereby a second deviant immediately following the first was presented. They subsequently calculated MMN(*D*_2_, *S*_5_, *t*) and found that it had smaller amplitude than MMN(*D*_1_, *S*_5_, *t*). In addition, they found that MMN(*S*_1_, *S*_5_, *t*) in this sequence had larger amplitude than MMN(*S*_1_, *S*_5_, *t*) from the sequence with only isolated single D stimuli. Our simulations agree with these experimental findings, as illustrated in Figure 10D, where the simulated MMN(*D*_2_, *S*_5_, *t*) is smaller than MMN(*D*_1_, *S*_5_, *t*) from Figure 10C, and the MMN(*S*_1_, *S*_5_, *t*) is larger in amplitude than MMN(*S*_1_, *S*_5_, *t*) from the previous sequence with occasional isolated deviants, shown in Figure 10C.

The authors of the experimental study interpreted the reduced-amplitude MMN(*D*_2_, *S*_5_, *t*) and the increased-amplitude MMN(*S*_1_, *S*_5_, *t*), relative to the corresponding cases with a single deviant, as evidence of the involvement of “neuronal models.” However, the agreement seen between their findings and the model simulations in this section suggest that adaptation can account for these findings. Again we suggest that the “neuronal models” posited in these early studies correspond to adaptation of the relevant cortical regions associated with each stimulus, at least to a first approximation. In favor of this is the way that these “neuronal models” appear to be strengthened by repeated stimuli (Sams et al., 1984). The present adaptation process naturally accounts for this by the fact that repeated stimuli drive greater adaptation, resulting in a larger mismatch when followed by a different stimulus.

#### 3.4.3. Sequences of Identical Stimuli With Different ISIs

Here we consider streams of identical stimuli in order to probe how adaptation and the resulting *S*_∞_ and MMN depend on the ISI. We also compare the model with an early study that looked at the ERs to tone-only sequences of infrequent stimuli (Näätänen et al., 1989a). Due to the development of the *S*_∞_ and *D* responses illustrated in section 3.3, an ISI on the order of ≈ 0.5 − 1 s should give rise to an adapted *S* response, where the number of repeated stimuli required for *S* to approach its limiting form *S*_∞_ depends on the ISI. As shown in section 3.3, for an ISI of 1 s, approximately 5 stimuli are required to approximate *S*_∞_.

**Figure 11:**
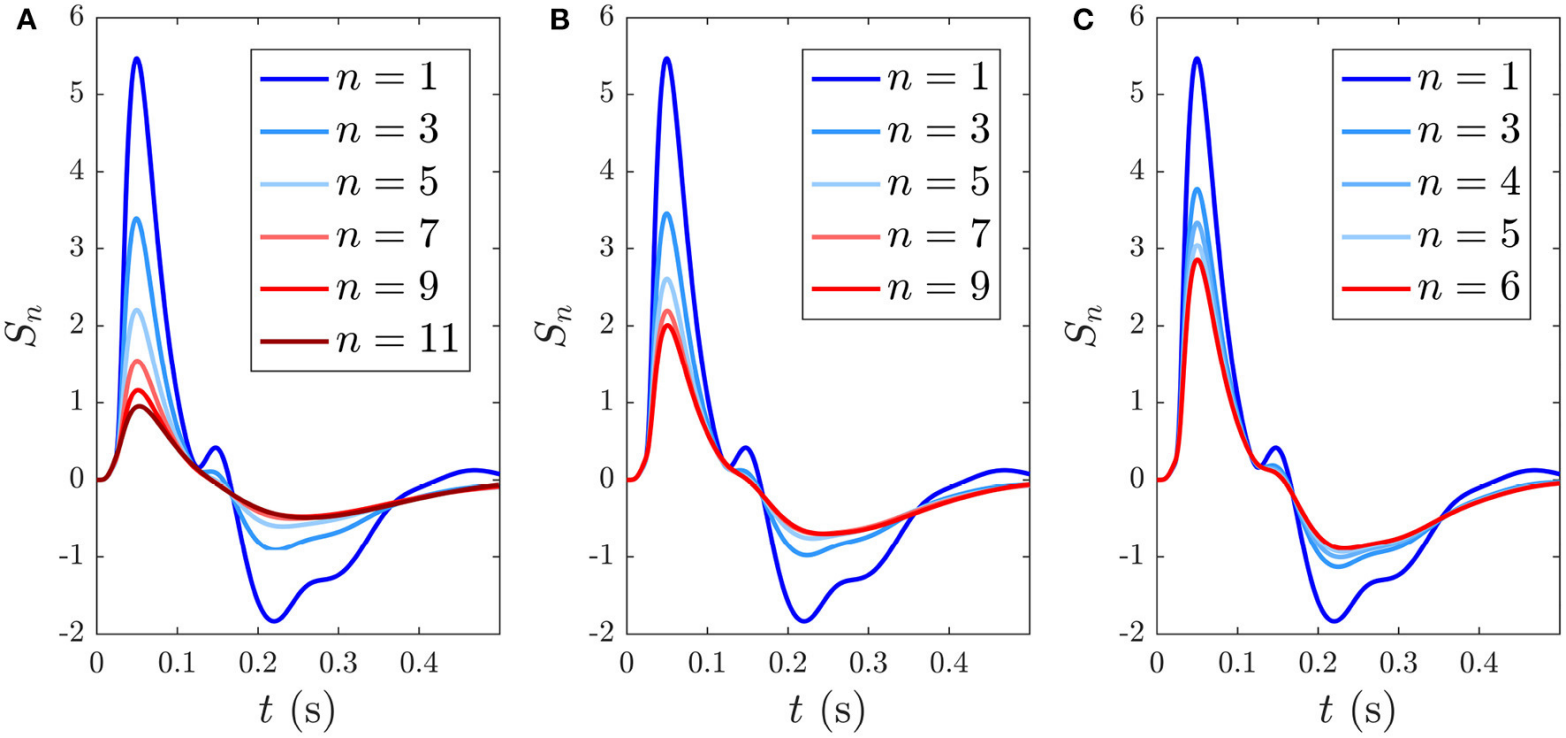
Development of the *S*_∞_ response during consecutive stimuli spaced at varying ISIs, with stimulus number indicated in the legends. **(A)** *S*_*n*_(*t*) for *n* = 1, …, 11 with a 0.5 s ISI. **(B)** *S*_*n*_(*t*) for *n* = 1, …, 9 with a 0.6 s ISI. **(C)** *S*_*n*_(*t*) for *n* = 1, …, 6 with a 0.7 s ISI.

**Figure 12:**
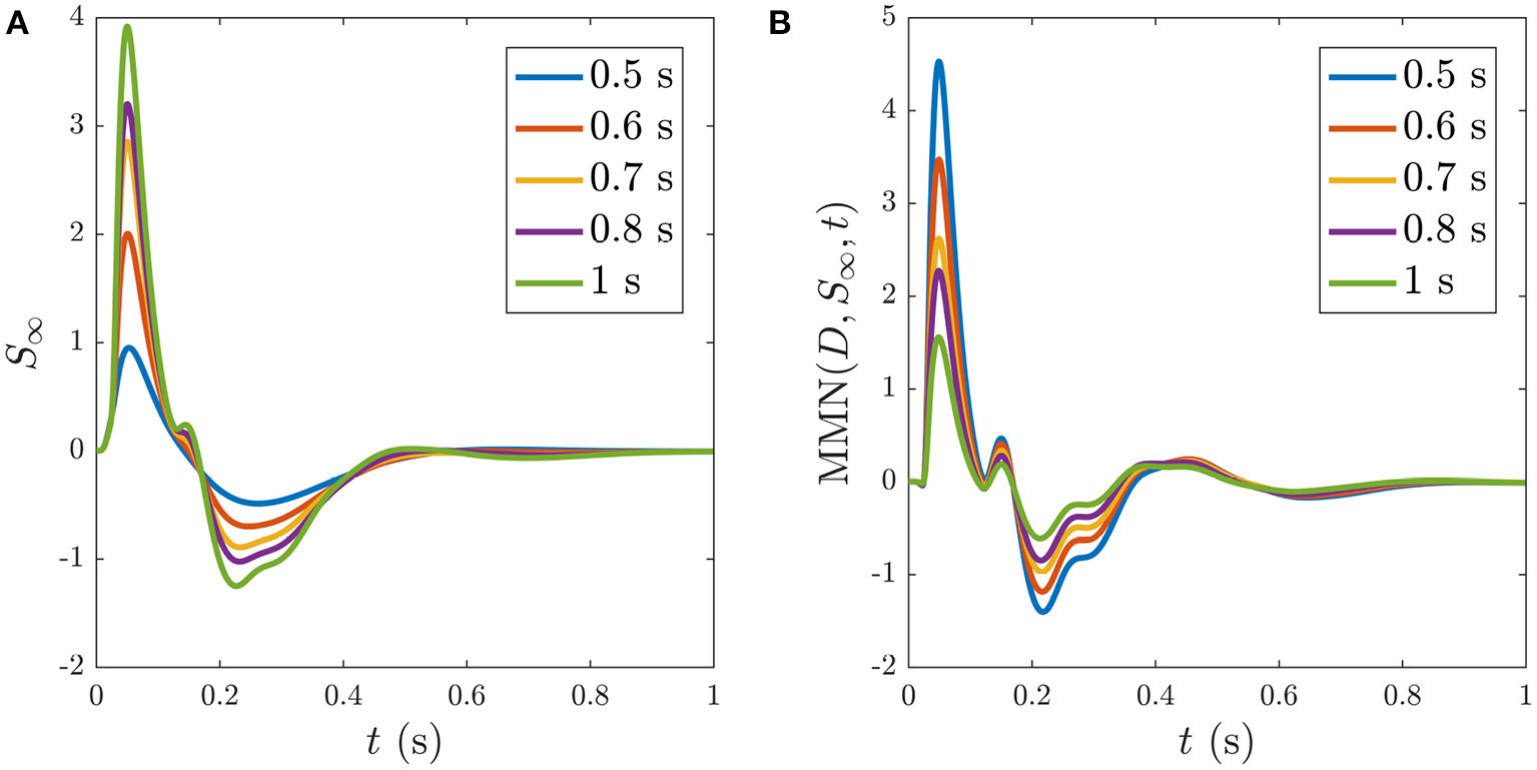
Dependence of *S*_∞_ on the ISI, with ISI indicated in the legends. **(A)** *S*_∞_ for ISIs of: 0.5, 0.6, 0.7, 0.8, and 1 s, as indicated in the legend. **(B)** The corresponding MMN(*D, S*_∞_, *t*) for each of the cases in **(A)**.

Reducing the ISI increases the number of stimuli that occur during the ~ 5 s window before *S*_∞_ is reached, as illustrated in Figure 11, which shows the development of *S*_∞_ due to multiple successive stimuli at varying ISIs; the *S*_∞_ parameters are thereby pushed further from the P state and its profile is modified accordingly. Figure 11A shows how *S*_∞_ is reached only after 11 stimuli spaced at an ISI of 0.5 ms, whereas Figures 11B,C show that *S*_∞_ is reached only after 9 and 6 stimuli spaced at ISIs of 0.6 and 0.7 ms, respectively. The adapted *S*_∞_ response therefore depends on the ISI. This is illustrated by simulating sequences of responses at variable ISIs and plotting the limiting form *S*_∞_ of each sequence vs. ISI, shown in Figure 12A along with the corresponding MMN(*D, S*_∞_, *t*) in Figure 12B. We see that as the ISI decreases from 1 to 0.5 s, the *S*_∞_ response curve exhibits smaller N1 and P2 amplitudes and the N2 component, which was small relative to the N1 peak in the 1 s ISI case, vanishes completely. Furthermore, as the ISI decreases the MMN amplitude increases, which agrees with experimental findings of increased MMN amplitudes at shorter ISIs (Ford and Hillyard, 1981; Nordby et al., 1988; Näätänen et al., 1993).

#### 3.4.4. Tone-Only Sequence

In our model completely discriminable stimuli do not affect one another's responses via adaptation, as expressed via Equation (51). As a result, streams of identical D stimuli result in identical streams of *D* responses, resembling those shown in Figure 6B, regardless of whether any fully discriminable S stimuli are presented in between.

In contrast to our pure-adaptation prediction, experiments have found that ERs to the infrequent stimuli alone (tone-only sequence) differed from the case where more frequent S stimuli occurred in between. Specifically, the tone-only responses did not exhibit a negative deflection overlapping the N1 and P2 features, but rather exhibited a larger N1 deflection (Näätänen et al., 1989a). This implies that effects other than adaptation of distinct cortical regions, such as higher-order processes and memory effects, play an important role in the distinction between these two cases. The present work enables the effects of adaptation to be separated from the other contributions to allow the latter to be focused on more specifically.

#### 3.4.5. Well-Separated Trains of Stimuli

We now investigate an experiment that specifically invoked higher-order processing as a contributing factor to the MMN, in order to tease apart how much can be explained by adaptation alone. The experiment (Cowan et al., 1993) investigated the links between MMN and memory representation by setting out to measure if a long-term or “silent” memory representation (longer than the typical decay rate of the *S*_∞_ response) of a given S stimulus persists over a long interval between two well-separated trains of S stimuli such that a D stimulus occurring in the second position of the second train elicits an MMN. Because the inter-train interval was longer than the MMN decay time, they concluded that any MMN associated with the second-position D reflects the reactivation of a memory representation that became dormant during the inter-train interval and was reactivated by the first S stimulus of the new train (Cowan et al., 1993). Their findings confirmed the presence of an MMN associated with the second-position D and led them to interpret this as evidence for such memory formation, inactivation, and reactivation.

The experimental procedure involved placing a D stimulus in position 1, 2, 4, 6, or 8 of a nine-item train of standards at an ISI of 610 ms between tones within a single train and an inter-train interval of 11 − 15 s (Cowan et al., 1993). We use a superscript *n* to label the position of the D stimuli within the 9-element train such that the above cases can be distinguished as $D^{1}$, $D^{2}$, $D^{4}$, $D^{8}$, and $D^{8}$. The authors found that stimuli at positions $D^{n\geq 2}$ were sufficient to yield an MMN (Cowan et al., 1993).

**Figure 13:**
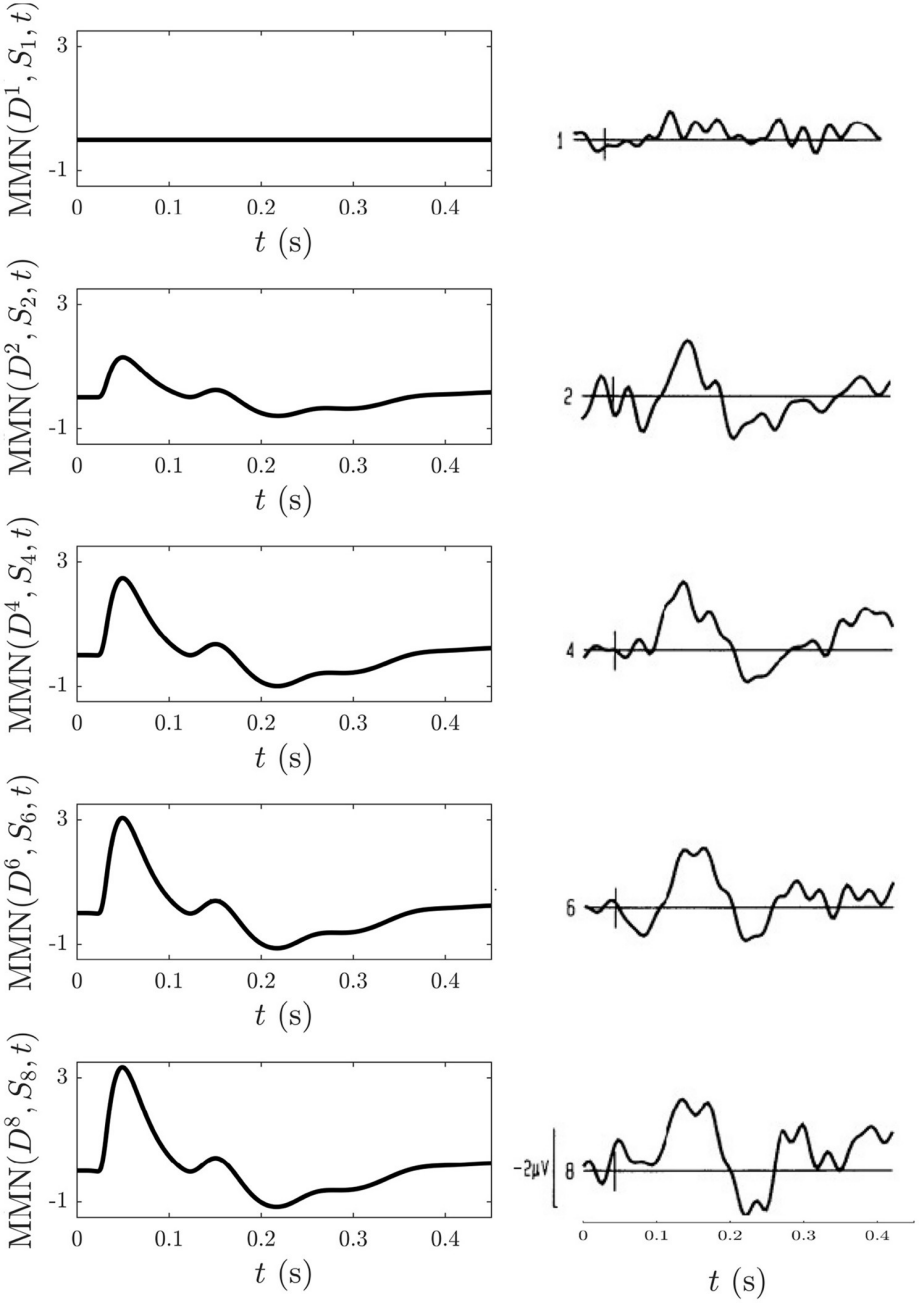
Comparison of model simulations and experimental findings (Cowan et al., 1993) discussed in section 3.4.5. Simulation of the MMN$\left(D^{n},S_{n},t\right)$ for *n* = 1, 2, 4, 6, 8 is shown on the left and the corresponding experimental MMN curves adapted from Cowan et al. (1993) on the right. The small vertical mark in each experimental frame indicates the stimulus time and the first curve gives an indication of noise levels.

To model this experiment we simulate the following blocks of trains: an S-only train: (9S), and nine-element trains of mostly S stimuli with D stimuli placed at positions listed above: D(8S), (1S)D(7S), (3S)D(5S), (5S)D(3S), and (7S)D(1S). The long inter-train intervals mean that there are no cumulative effects from the adaptation occurring in each train that last until the next train and thus that such effects can be disregarded, allowing each train to be studied in isolation. To follow what was measured experimentally, the MMN corresponding to the *D* responses at the positions listed above with respect to the corresponding *S* response at the same position in the S-only train, MMN$\left(D^{n},S_{n},t\right)$ was calculated and is shown Figure 13 alongside the corresponding experimental MMN adapted from Cowan et al. (1993).

We find that MMN$\left(D^{n},S_{n},t\right)$ is zero at *n* = 1, because *D*_1_ = *S*_1_, and that it increases with *n* because adaptation effects alone are enough for $D^{2}$ to elicit an MMN in the above trains without the need to invoke memory formation and representation processes. The model detection of a nonzero MMN$\left(D^{2},S_{2},t\right)$ reflects the fact that the *S*_2_ response in a stream of standards has already adapted significantly enough to cause a mismatch with the subsequent *D* response. The extent of the adaptation depends on the ISI, as shown in section 3.4.3. Other similarities between the model MMN$\left(D^{n},S_{n},t\right)$ and experiment include the presence of N1 and N2 deflections as well as P2 and P3 deflections emerging as *n* increases, as well as an increase of the N1 deflection as *n* increases. Experimentally, as *n* increases the N2 deflection approaches that of N1, whereas their relative amplitudes remain unchanged in the model simulations. This could reflect an effect of the memory representation process that was conjectured to be involved (Cowan et al., 1993), especially because considerable experimentation with changing the model adaptation parameters found the N2 peak to consistently remain smaller in amplitude compared to the N1 peak.

Overall, the level of agreement between model simulations and experiment for the above sequences suggests that adaptation can explain key features of the experiment that were previously assumed to be the result of higher order processes (Cowan et al., 1993). However, such processes are likely to be needed to account for the remaining differences between the model and experimental findings concerning the relative amplitudes of the N1 and N2 deflections.

## 4. Summary and Discussion

We have modeled and analyzed sequences of auditory evoked responses, used in human cognitive studies, by means of a physiologically based neural field theory whose predictions have previously reproduced a wide range of experimental data on brain activity and connectivity, as mentioned in the Introduction. To do so, the theory was generalized to include corticothalamic adaptation to repeated stimuli arriving at a given point on the tonotopic map. Repeated stimulation within the 5–10 s lifetime of adaptation leads to greater movement of corticothalamic gains away from the prestimulation baseline and contributes to standard responses evolving away from deviants.

The central aim of the work is to provide a means of calculating the response to arbitrary sequences of discriminable stimuli in order to determine how much of the dynamics can be accounted for by adaptation and how much might be ascribable to higher-order top-down memory-related stimulus-comparison processes—a long-running controversy in the field. This accords with Occam's Razor, which dictates that one should first determine how much can be explained by low-level processes such as adaptation in thalamus and primary auditory cortex before invoking higher-order aspects. However, we stress that the latter processes are certainly relevant in many contexts, especially those involving long-term memory or comparison of abstract stimulus features.

This work provides a quantitative bridge between biophysical analysis of brain activity and electrophysiological measurements of human cognitive processes, in more detail than has previously been possible. Tools based on this approach should help to clarify the relative roles of low-level adaptive processes and high-level feedbacks in determining evoked responses in various situations.

The main outcomes are:

- A corticothalamic NFT model of the medial geniculate nucleus of the thalamus and the primary auditory cortex was formulated in which ERs are viewed as impulse responses, with evoked changes occurring both directly in the activity and indirectly via the system gains, resulting in a bilinear response that we treated via perturbation theory. Long-timescale adaptation of gains was also incorporated for the first time.
- The dynamical building blocks of ERs are damped oscillations at natural resonant frequencies, usually with deflections of both polarities. This is in contrast to the traditional notion of ER components with fixed polarities and timings and in accord with temporal shifts and inversions of some features during development (Kerr et al., 2010).
- Adaptation changes both amplitudes and timings of ER waveforms. This invalidates common assertions that adaptation can change only amplitudes of traditional ER components with fixed timings.
- The MMN is a mathematical entity that is constructed by subtracting one response from another. Most commonly the response to a common standard is subtracted from the response to a rare deviant, but there is no unique definition. However, if the parts of ERs that are due to adaptation can be identified and shown to be insufficient to account for the differences between the two responses (i.e., for their MMN), the remainder may well be attributable to higher-level processes.
- Our generalized notation for the MMN between two responses—e.g., MMN(*S*_3_, *S*_5_, *t*) for the third standard relative to the fifth—highlights the implausibility of there being a separate dedicated set of neurons that generate an MMN for every possible comparison that might be conceived of by experimenters. This is thrown into stark relief when one notes that any pair of responses whatsoever can be used to define an MMN—even responses in different sensory areas at very different times.
- Identification of higher-order processing and other effects is facilitated by the model. This is because one model must be able to account for a given subject's responses to arbitrary stimulus sequences—ideally with little or no change in parameters so long as the subject's physiological state is unchanged. Hence, once parameters have been calibrated on sequences of identical stimuli at various ISIs, for example, they should yield the responses to arbitrary stimulus sequences, as far as adaptive changes go. Further differences can then be explored as potentially being due to other mechanisms.
- Deviant ERs start from a point nearer the corticothalamic baseline than standard ERs, which begin from a point that adaptation has driven away from the pre-stimulation state. Corticothalamic feedforwards and feedbacks change in strength with adaptation, with the largest changes found to be in gains involving the cortex, as summarized in Table 2. This is broadly consistent with theories such as predictive coding in which top-down predictions are compared with bottom-up signals and the system adapts to reduce the discrepancy (Garrido et al., 2009b).
- In oddball paradigms, the model accounts naturally for (a) the difference between *S* and *D* responses, (b) the effects of consecutive D stimuli on subsequent *S* and *D* responses, (c) the effect of the position of the stimulus in a long train, and (d) the development of *S* responses (starting as identical with *D* ones) with repeated presentation, and their decay after a stimulus-free interval.
- Some aspects of ERs have not been accounted for by adaptation alone, which points to their likely dependence on higher-order processes and feedbacks. These include tone-only sequences which provoke the same model *D* responses regardless of whether discriminable *S* responses occur in between, which is not in accord with experimentally observed differences between the two cases. Likewise, differences between well-separated stimulus trains with deviants in different positions can be partly explained by adaptation effects, but late structure has not been fully reproduced and needs further investigation. In this context, we again stress that our aim is not to account for all ER features by adaptation, but to determine which features can be explained in this manner so as to focus attention more sharply on those that are produced by other mechanisms.

Overall, we have shown that adaptation can account for many but not all features of ERs in various stimulus sequences. This both highlights the role of such processes in the initial corticothalamic stages of signal processing and cognition and points the way to focus on higher-order aspects, especially in humans. The formulation in terms of resonances and gains makes immediate links to control-systems interpretations that tie the results to the dynamics of prediction and unconscious attention behind many cognitive processes (Babaie-Janvier and Robinson, 2018, 2019). More generally, the NFT used has accounted for a wide variety of normal and abnormal brain activity and connectivity phenomena, as mentioned in the Introduction, so ER dynamics is thereby also integrated into this broader landscape.

The present work provides a starting point for quantitative exploration of the role of adaptation in the host of ER sequences that have been studied in the literature discussed in the Introduction. This would include analysis of ERs to sequences of stimuli that are not fully distinguishable, omitted tones, tones of variable frequency, duration, or amplitude, and other variants. For optimal outcomes the model should be calibrated for individual subjects on simple oddball sequences, then used to predict their responses to more complex stimulus sequences—something that has previously been done when applying NFT to a range of other phenomena mentioned in the Introduction. A key advantage of NFT is that its parameters are closely tied the physiology, so links to underlying biophysics are more direct and easier to make than via phenomenological component analysis.

Many further extensions and applications of the model can be made. A key generalization needed to better probe ERs is to include spatial aspects of the tonotopic map and the responses to enable comparison with observations of ER topography. Some such work has been done on evoked responses using NFT, albeit without adaptation (Mukta et al., 2019; Robinson et al., 2019) and the work here will enable it to be generalized by appropriately modifying the response functions and including the spatial structure of natural modes of brain activity. Application to ERs that involve other auditory features (e.g., interaural delays and directionality), or other sensory modalities, is also an obvious direction for future work because the present formulation is certainly not limited to auditory systems. Similarly, one could apply this approach to evoked responses in nonhuman animals, although the parameters would need to be recalibrated in that case. It is also worth noting that the stimuli used do not have to be impulsive—replacement of a delta-function input by a periodic drive enables steady-state evoked responses to be studied, as has previously been done in the absence of adaptation (Robinson et al., 2008).

## Data Availability Statement

The original contributions presented in the study are included in the article/Supplementary Material, further inquiries can be directed to the corresponding author/s.

## Author Contributions

PR conceived the project and performed the analytic work. NG and TB-J performed the coding, the numerical calculations, and their analysis. All authors drafted the respective sections of the MS and collaborated to produce the final version.

## Conflict of Interest

The authors declare that the research was conducted in the absence of any commercial or financial relationships that could be construed as a potential conflict of interest.

## Publisher's Note

All claims expressed in this article are solely those of the authors and do not necessarily represent those of their affiliated organizations, or those of the publisher, the editors and the reviewers. Any product that may be evaluated in this article, or claim that may be made by its manufacturer, is not guaranteed or endorsed by the publisher.
